# Aroma of Sherry Products: A Review

**DOI:** 10.3390/foods10040753

**Published:** 2021-04-01

**Authors:** Enrique Durán-Guerrero, Remedios Castro, María de Valme García-Moreno, María del Carmen Rodríguez-Dodero, Mónica Schwarz, Dominico Guillén-Sánchez

**Affiliations:** 1 Analytical Chemistry Department, Faculty of Sciences-IVAGRO, Agrifood Campus of International Excellence (ceiA3), Campus Universitario de Puerto Real, University of Cadiz, s/n, Puerto Real, 11510 Cadiz, Spain; remedios.castro@uca.es (R.C.); valme.garcia@uca.es (M.d.V.G.-M.); maricarmen.dodero@uca.es (M.d.C.R.-D.); dominico.guillen@uca.es (D.G.-S.); 2 “Salus Infirmorum” Faculty of Nursing, University of Cadiz, 11001 Cadiz, Spain; monica.schwarz@uca.es; 3 Nutrition and Bromatology Area, Faculty of Medicine, University of Cadiz, Plaza Falla, 9, 11003 Cadiz, Spain

**Keywords:** Sherry, wine, vinegar, brandy, aroma

## Abstract

Jerez (Sherry) is a well-known wine-producing region located in southern Spain, where world-renowned oenological products such as wines, vinegars, and brandies are produced. There are several factors that provide characteristic physical, chemical, and sensory properties to the oenological products obtained in this Sherry region: the climate in the area with hot summers, mild winters, and with limited rainfall; the raw material used consisting on Palomino Fino, Moscatel, and Pedro Ximénez white grape varieties; the special vinification with fortified wines; and aging techniques such as a dynamic system of biological or oxidative aging. These special organoleptic characteristics are responsible for, among others, the aromatic profile of the wines, vinegars and brandies from the area, which explains why this is a subject that has been extensively researched over the years. This bibliographic review aims to compile the different scientific contributions that have been found to date, in relation with the aroma of the oenological products from the Sherry area (dry wines, sweet wines, vinegars, and brandies). We have mainly focused on the different analytical methodologies used and on the main analytes of interest.

## 1. Introduction

The winemaking tradition in the agricultural areas within the Jerez (Sherry) region dates far back in time. This is an eminent wine-producing region located in the south of Spain, surrounded by mountains and coastal lands that condition the climate in the area, which together with its particular aging methods, are determinant to attain the highly desirable organoleptic characteristics of its oenological products [1]. Worldwide renowned oenological products such as wines, vinegars, and brandies are the result of this unique combination of factors.

Sherry wines are considered among the most highly appreciated products in the world of oenology [2]. Diversity is undoubtedly one of the distinctive features of Sherry’s identity, where just three grape varieties (Palomino, Moscatel, and Pedro Ximénez) give rise to different wines that clearly differ in terms of color, aroma, flavor, and texture depending on their elaboration process. [3].

Those wines that are subjected exclusively to biological aging—i.e., those which are protected from any direct contact with the air by the natural flor velum—retain their initial color, and display a series of distinctive aromatic and gustatory notes derived from the yeasts that form that essential flor velum [4]. On the other hand, other Sherry wines are aged by oxidative or physicochemical means, in direct contact with the oxygen in the air. These gradually acquire a darker hue, and exhibit more complex aromas and flavors [5].

Furthermore, the type of fermentation, which can be either complete or partial allows the production of highly dry wines (fortified wines) or extraordinarily sweet wines (natural sweet wines). By mixing these two types in different proportions, new wines with varying levels of sweetness (liqueur fortified wines) are also obtained [6,7].

With regard to Sherry vinegars, these are obtained from the grapes grown in the local vineyards. The authorized grape varieties for the production of Sherry vinegar are the same that those employed for Sherry wine. The Sherry vinegar production process basically consists in the acetic fermentation of local wines, as a result of the transformation of alcohol in acetic acid by acetic bacteria (*Mycoderma aceti*) and its subsequent aging in wooden casks. The final product presents a color between old gold and mahogany, with an intense aroma, lightly alcoholic, with notes of wine and wood predominating, and a pleasant taste, despite the acidity, with a long aftertaste [8,9].

On the other hand, Sherry Brandy is the product resulting from the distillation of wines (mainly Airén and Palomino ones) and its subsequent aging to confer the final product its distinctive organoleptic qualities [10].

All these products share in common a singular and dynamic aging process that is characteristic of the Sherry area: ‘Criaderas y Solera’. This aging process uses oak casks, generally American oak (*Quercus alba*), that may vary between 250 and 600 L volume depending on the product to be obtained. The porosity of the American oak is ideal to allow the contact of the aging product with the oxygen in the air, thus facilitating its oxidation and favoring the aging process. The evolution of all the product physicochemical parameters is largely due to the impact of wood on the aging process. In fact, wood is a definite determinant of the organoleptic properties achieved by all the Sherry oenological products [5,11]. Moreover, the high level of aromatic content of these Sherry products is also influenced by the high level of aromatic composition of the American oak, compared to other types of oaks, such as French oak (*Quercus petraea, Quercus robur*).

During the aging phase in the winemaking process, the capacity of the wood to release certain compounds is essential and will vary according to the size and age (previous uses) of the cask. Thus, the smaller the cask size, the greater the wood surface in contact with the liquid. In this sense, the use of small barrels is not always convenient, since the effect of the wood on the final product could be greater than desirable [12]. Based on experience, 500–600 L barrels seem to be the most appropriate size for the aging of Sherry products, since they provide the ideal balance between wood surface and content volume.

Another characteristic of these wines is that they are aged in preconditioned casks, i.e., casks that have previously contained sherry wine. They are known as *“barricas envinadas”* (casks in which Sherry wine has been aged). This significantly contribute to providing these products with different nuances depending on the type of preconditioning undergone by the casks [13].

**Figure 1:**
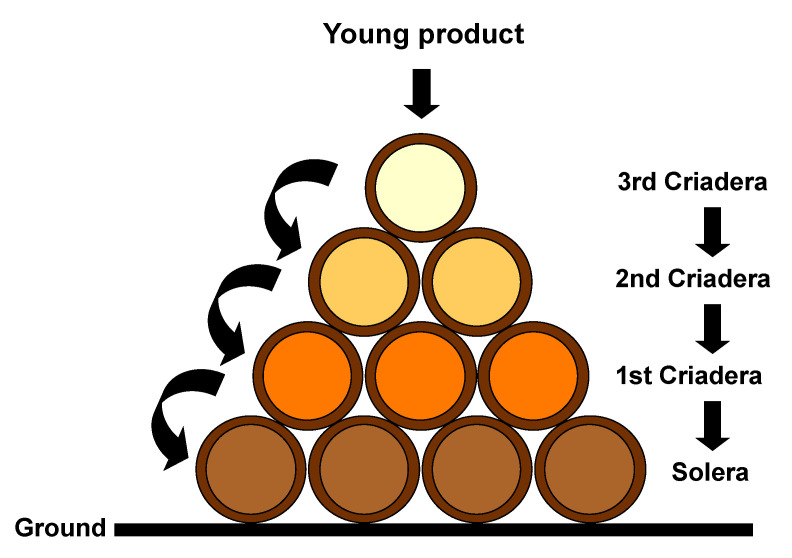
Criaderas y Solera aging method.

The aforementioned ‘Criaderas y Solera’ aging method could be defined as a dynamic aging process, as opposed to the static aging by vintages. In the latter system, the oenological product to be aged remains in the same barrel during the entire aging period, while in the Criaderas y Solera method, however, the oenological product is stored in casks classified into groups, known as ‘scales’, according to the age of the product that they contain. The scale that contains the oldest oenological product is called ‘solera’ and it is located at ground level. This is topped, according to its younger age, by the first criadera, the second, the third and so on (Figure 1). A small amount of the product, which must be the same from each of the casks that make up the solera, is extracted for bottling and distribution. The resulting empty space is replenished with the equivalent volume of the oenological product from the first criadera. The same procedure is applied to the first and second criadera, which are refilled with the product from the corresponding topping criadera. In this way, a uniform product is obtained in terms of flavor, aroma and color. The same organoleptic characteristics are obtained, since the amount of refilling product is rather reduced in comparison with the larger amount of product in the receiving cask. Thus, the small amount of product added to the cask acquires the characteristics of the predominant older product it is mixed with [14,15].

Because of this peculiar aging process, it is quite difficult to estimate the exact age of the oenological product, so it is usually referred to as average age. This parameter is defined as the ratio between the total volume of product in the system and the annual volume that is taken out for its commercialization. Depending on their average age, they will be classified into different categories, which will exhibit different characteristics, depending on the original oenological matrix that was used (wine, vinegar, or Brandy).

All the aforementioned features in the elaboration of Sherry products provide them with their own qualities that will constitute their seal of quality. Thus, such characteristics like polyphenolic compounds content [10,16,17,18], chromatic attributes [17,19,20], organic acids [13,17], or sugars contents [14] have been suggested to be determinant parameters regarding the ultimate quality of Sherry wine, vinegar, or brandy.

The aroma of oenological products, in general, represents an important determinant of their quality, and there are numerous studies that support this point [21,22,23,24]. Although not all volatile compounds contribute to aroma perception [25], the study of aromatic profile is still of major importance, since the acceptance of the final product by the consumer depends on them to a great extent [26]. Consequently, in recent years, significant technological advances have been made in terms of extraction methods and the subsequent analysis of these compounds [27,28]. In parallel, sensory analysis has been consolidated as an essential tool to perform a complete investigation that covers all the aspects related to aroma. An increasing number of studies propose sensory analysis as a crucial tool to determine the quality of the final product [29]. Moreover, a recent study by Cruces-Montes et al. [30] presented the perception of the attributes of Sherry wine and its consumption in young people in the south of Spain. Their results showed that the consumption of Sherry wine was recognized to different dimensions, and flavor was especially important for some types of Sherry wine.

**Figure 2:**
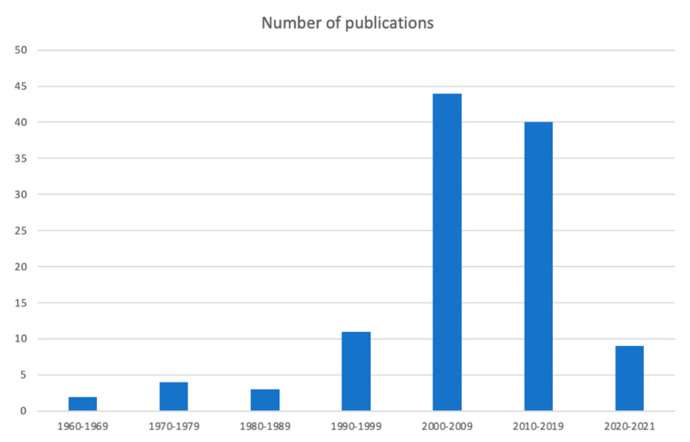
Number of publications addressing the aroma of Sherry oenological products. Source: Scopus.

Figure 2 shows the growing progression in the number of studies that address the subject of aroma in the typical products from Jerez (Sherry) area: wine, vinegar, and brandy. This rising number of studies and publications is explained by the importance of the content of volatile compounds regarding the aroma of wine products, as well as by the socioeconomic relevance of these products in the region. Also, the evolution of analytical technologies and their innovations contribute for the increment of this kind of studies. On these bases, we have considered the importance of a literature review that would cover the most prominent aspects associated to this tandem: aroma and Sherry oenological products.

## 2. Study of the Aroma of Dry Sherry Wines

The aromatic content of Sherry wines and, in particular, that of Fino wines, has been extensively studied by employing the analytical methods previously described. Table 1 shows the main aromatic compounds detected in Fino, Amontillado, and Oloroso Sherry wines, together with the bibliographic references where these compounds are mentioned. Sensory descriptors and concentration ranges also appear in Table 1.

All types of dry Sherry wines are produced from the same grape, ‘Palomino fino’, and it is the subsequent elaboration of the product (biological or oxidative aging), the main responsible of obtaining wines with different organoleptic characteristics. Also, the extracted wood components induce these changes. Therefore, it is mainly the aging process that will determine the differences between the three types of dry wines: Fino, Oloroso, and Amontillado. Thus, the aroma of Fino wines will be conditioned by the flor velum yeast, which, in addition to shielding the wine from oxygen, will contribute with a series of compounds derived from its metabolism. At the other end, we have the Oloroso wine that undergoes oxidative aging and contains higher levels of alcohol, so that during this aging stage the compounds that were initially present in the wine aroma will evolve due to oxidation, esterification, and other reactions. Finally, Amontillado wines undergo a first stage of biological aging and then an oxidative one [5].

A large number of volatile compounds are common to all of them, including acetaldehyde, acetoin, eugenol, and 1,1-diethoxyethane, among others. Acetaldehyde may come from different sources, although it appears particularly as a secondary product resulting from the aerobic metabolism of the flor velum yeasts responsible for the biological aging process [40,41]. This compound is also the precursor of a large number of other compounds that are involved in the aroma of Sherry wines, either as a result of biological or oxidative aging. In particular, it is the precursor of 1,1-diethoxyethane, one of the main acetals in Sherry wines, which is formed through chemical and biochemical reaction with ethanol [42]. This compound contributes to the fruity aromas and balsamic notes of these wines.

Acetoin is one of the other acetaldehyde-derived compounds with aromatic significance in Sherry wines. This compound is preferentially formed by a condensation reaction of two acetaldehyde molecules [42]. Acetoin is one of the compounds responsible for the bitter notes of Fino wines. The reduction of the acetoin gives rise to 2,3-butanediol, another aromatic compound involved in the aroma of Sherry wines.

The reaction between acetaldehyde and α-ketobutyric acid during the anaerobic metabolism of the yeasts in the flor velum gives rise to sotolon. This compound has a high impact on the aroma of these wines, particularly in the nutty, curry, and cotton candy notes that are present in all the Sherry wines [42].

It should be noted that Sherry wines from exclusively biological aging—i.e., Fino wines—have a particularly high acetaldehyde content, which is actually attributable to their biological aging. This compound is not only responsible for the sharp character of Fino wines’ aroma, but also contributes enriches it with the notes of overripe or ripe apples [33,35,43] that are inherent to this wine.

According to the bibliography, other major volatile compounds to be found in the wine are isoamyl alcohols, ethyl lactate, and 1,1-diethoxyethane (Table 1). A certain number of volatile compounds clearly differentiate Fino wines from other types of Sherry wines, among them E-3-hexenol, Z-3-hexenol, γ-decalactone, terpinen-4-ol, Z-nerolidol, farnesol, and octanal. This suggests that their origin may be linked to the biological aging process that characterizes this wine, and that they do not remain as part of the composition of other wine types, like Amontillado, which undergoes a subsequent oxidative aging procedure.

Other aromatic compounds that play a significant role in the aroma of biologically aged wines are β-citronellol and β-ionone. These compounds are responsible for the citrus and balsamic notes in the aroma of these wines, although they are present at concentration levels of μg/L (Table 1). Other compounds that also stand out are phenethyl octanoate, ethyl palmitate, nerol, propyl butanoate, and ethyl myristate. All of these compounds have been detected in both Fino and Amontillado wines (Table 1).

Amontillado wines, which are obtained through an initial biological aging stage and a subsequent oxidative process as above mentioned, exhibit certain characteristics of their own. For example, they do not contain ethyl benzoate in their composition; they are the only types of Sherry wines that present detectable concentrations of isobutyl isobutanoate (0.066 mg/L) and isoamyl laurate (0.357 mg/L), and present lower concentration levels of 1,1-diethoxyethane, isobutanol, and phenethyl alcohol, while their levels of E-nerolidol are higher with respect to that in Fino or Oloroso wines [32]. The main volatile compounds that can be found in Amontillado wines are ethyl lactate, acetaldehyde, isoamyl alcohols, diethyl succinate, and ethyl acetate, all of them at levels of concentration of dozens or even hundreds of mg/L (Table 1). It has long been known that oxidative aging results in a higher concentration of esterified compounds in Amontillado wines, since their greater concentration of ethanol results in evident increment in ethyl lactate and ethyl acetate concentrations during the aging phase [32]. However, the compound that contributes the most to the aroma of Amontillado wines is ethyl octanoate, that is usually present at concentrations below 1 mg/L [37], followed by ethyl butanoate, eugenol, ethyl isobutanoate, and sotolon, which maintain their relative contributions to the wine aroma throughout the period of oxidative aging, even though their concentrations increase with time. It is precisely this second aging stage, the oxidative one, which confers Amontillado wines their main odorant characteristics.

Considerable levels of acetaldehyde are also found in Oloroso and Amontillado wines, although in lower concentrations than in Fino wines (around five times lower) [42]. The most abundant compounds in Oloroso wines are isoamyl alcohols, ethyl lactate, ethyl acetate, acetaldehyde, and diethyl succinate. Other compounds such as ethyl butyrate, ethyl caproate, ethyl decanoate, ethyl isovalerate, ethyl valerate, guaiacol, hexyl acetate, hexyl hexanoate, hexyl lactate, methyl acetate, 2-methylbutan-1-ol, methyleugenol, β-methyl-γ-octalactone, nonanoic acid, 2-phenylethanol, and 2-phenylethanol acetate tend to be more characteristic of Oloroso wines, and are not found either in Amontillado or Fino wines.

The narrow correlation between the aromatic composition of Sherry wines and the type of cask wood as well as the degree of toasting of the wood has already been studied [39]. The wines aged in French oak and chestnut casks undergo greater changes in their volatile compound composition during the oxidative aging process. American and Spanish oak, on the other hand, modify to a lesser degree the volatile compound profile of these wines during their aging. In relation to the wood toasting degree, it is the medium-toasted casks that produces the wines with the greatest volatile composition. These results are similar to those reported by other authors with regard to fortified and sweet wines aged in wood [44,45]. Eugenol and guaiacol are compounds derived from the degradation of lignin and their content increases during the aging in contact with wood. β-methyl-γ-octalactone was only identified in Oloroso wines aged in contact with oak wood, but not in those aged with chestnut. High concentrations of γ-butyrolactone were also determined in all the samples studied, similarly to those already reported by Hevia et al. [44]. Ethyl valerate, hexyl acetate, or ethyl octanoate (compounds that contribute with floral and fruity notes to the aroma of the wines) decreased with aging, except for the wines aged in French oak casks, which saw their concentration increased along with other compounds such as isobutyl acetate, ethyl valerate or isoamyl acetate.

## 3. Study of the Aroma of Natural Sweet Wines

According to the specifications in the Protected Denomination of Origin “Jerez-Xérès-Sherry” [6], Natural Sweet Wines are those produced using musts from very ripe or sun-dried grapes, generally of the Pedro Ximénez (PX) or Moscatel varieties. These musts, which are rich in sugars as a result of the raisining process, are only partially fermented in order to preserve most of their original sweetness. During this sweet vinification, the musts are fortified with wine alcohol as soon as the fermentation process starts, to reach a minimum alcohol content of 15% vol. The wines produced through this method are subsequently aged in direct contact with atmospheric oxygen, which favors a progressive aromatic concentration and increases their complexity while an intense color and a dense appearance is acquired, although with no negative impact on the typical freshness of these varieties. The alcohol content should range between 15° and 22° vol.

Table 2 presents the different volatile compounds determined in natural sweet wines, their sensory descriptors as well as the concentration ranges reported in the bibliographic references.

The volatile composition of a selection of sweet Andalusian PX and Moscatel wines was studied by Márquez et al. [52]. The major compounds identified included ethyl acetate, isoamyl alcohols, ethyl lactate, acetic acid, 2-furaldehyde, linalool, diethyl succinate, α-terpineol, and 2-phenylethanol. Both varietals presented elevated contents of isoamyl alcohols, ethyl acetate and ethyl lactate, fatty acids such as hexanoic, octanoic, and decanoic acids. Norisoprenoid 1,1,6-trimethyl-1 and 2 dihydro naphthalene (TDN) at low levels were also confirmed. Muscat presented very high concentrations of linalool, α-terpineol and limonene, and higher ones than PX in TDN. On the other hand, 2-furaldehyde and 5-methyl-2-furaldehyde were detected at significant levels in PX. With respect to PX, and according to the data provided by Campo et al. [56], who analyzed different types of dessert wines, PX also contains significant concentrations of 3-methylbutanal, phenylacetaldehyde, methional, sotolon, and the ethyl esters 2-, 3-, as well as 4-methylpentanoic acids, all of them with high aromatic activity. Nevertheless, the compounds that best differentiated the PX from the other wines were 3-methylbutanal, furfural, β-damascenone, ethyl cyclohexanoate, and sotolon.

The aromatic profile of the natural sweet wines from the Jerez-Xérès-Sherry Protected Designation of Origin (PDO) are the result of different contributions in the course of their production; from the grapes’ cultivation to the aging of the wine. It is necessary to clarify that, given that the musts obtained from raisined PX grapes fortified with wine alcohol from the neighboring production area of Montilla-Moriles PDO can be used, we have included in this bibliographic research the works that have also studied those musts.

**Figure 3:**
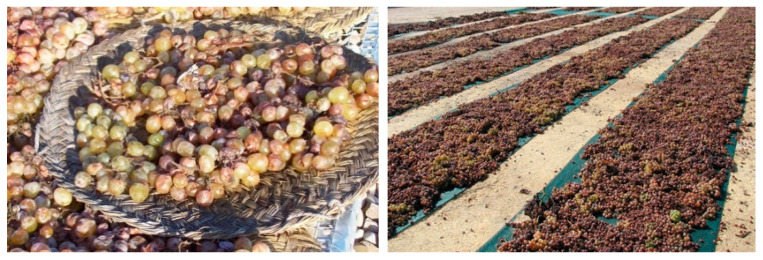
Traditional sun drying process for the raisining of the grapes.

While the sugar enrichment of the grapes can be achieved through the overripening of the grapes on the vines by twisting their stems without cutting them off, the traditional system in the Jerez (Sherry) region is the so called ‘asoleo’, which consists on drying the bunches of grapes in the sun for several days in order to partially dry or raisin the grapes (Figure 3).

A certain concentration of the compounds is to be expected, but Franco et al. [48], compared the aromatic profiles of sun-dried raisins and fresh grapes’ musts and were able to confirm the decrease in concentration of farnesol and of some 6-carbon alcohols and aldehydes responsible for herbaceous aromas (hexan-1-ol, (E)-hex-3-en-1-ol, (Z)-hex-3-en-1-ol, (E)-hex-2-en-1-ol, hexanal, and (E)-hex-2-enal). The authors attributed this reduction in specific compounds to the inactivation by exposure to light of the lipoxygenase enzymes responsible for the production of C6. They also detected very marked increments in the content of some other volatiles: isobutanol; benzyl alcohol; 2-phenylethanol; 5-methylfurfural; γ-butyrolactone, and γ-hexalactone, all of them related to the anaerobic metabolism of sugar, which encouraged the authors to suggest the promotion of this mechanism during the ‘asoleo’ traditional overripening system of the grapes, as it is known to occur in freshly harvested grapes [59]. In addition, high temperatures favor the formation of products derived from Maillard reactions which are responsible for roasted coffee or cocoa aroma notes. The complexity of these phenomena that affect the aromaticity of raisined grape must was analyzed by López de Lerma et al. [51]. They hypothesized that the criterion for determining the optimum raisining length of time perhaps should not be determined by aiming at a sugar concentration of around 400 g/L. In fact, they observed that some of the aromatic families of interest related to fruity and toasted notes started to decrease in concentration at an earlier stage, so they recommended reducing dehydration, and opted for rapid response tools such as the electronic nose to control the process. For Ruiz et al. [50] however, raisining consists of two stages: during the first 4 days, slight changes occur in the chemical and sensory aromatic profiles, and thereafter the raisins are substantially enriched in aromas.

**Figure 4:**
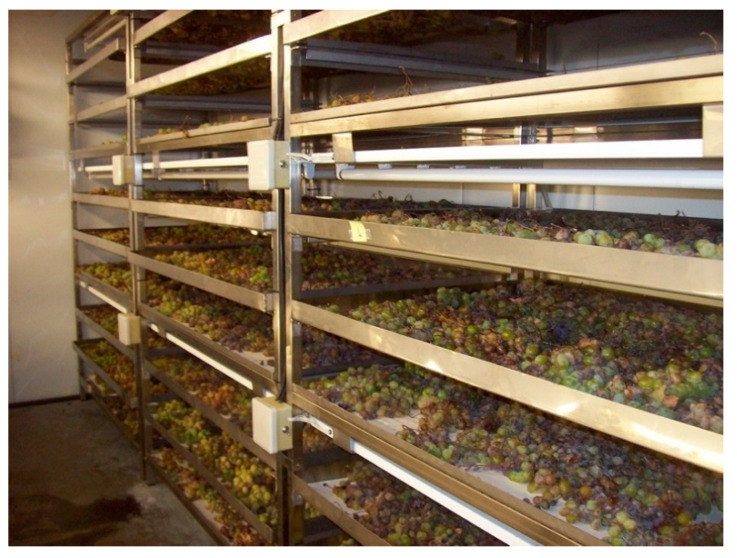
Climatic chamber with temperature and moisture control.

During the ‘asoleo’ traditional overripening system a number of risks are faced, such as the possibility of rain or nighttime moisture, which may result in a loss of quality due to fungal attacks [60]. Several researchers have studied an alternative of great interest such as the use of climatic chambers to keep the control on temperature and humidity conditions (Figure 4). This method would also allow the raisining stage to be shortened.

Ruiz et al. [50] compared the volatile compositions of raisined grape musts obtained by “asoleo” traditional overripening system or in a climatic chamber. The data obtained were processed as aroma values and grouped into aromatic families, according to their contribution to characteristic olfactory notes. The caramel note was the highest value in both cases (associated with increases in 3-hydroxy-2-butanone, known as acetoin, γ-butyrolactone, and 2,3-butanedione) and together with the floral note related to concentration increases in geranial, phenethyl acetate, phenethyl alcohol and farnesol, were perceived more clearly in the musts obtained from climatic chamber raisins. The same authors [46] analyzed the effect of chamber temperature and drying time on the PX grapes and a combination of 40 °C for 96 h was established as ideal to obtain more intense caramel and floral notes (mainly due to important increases in phenethyl alcohol) together with a characteristic and highly appreciated milky note associated to an increment in methylbutanoic acid. However, these results do not agree with those obtained by Serratosa et al. [61], who considered 50 °C as a better option that allowed them to obtain a must that was sensorially very similar to that produced by traditional raisining methods. Ruiz-Bejarano et al. [62] evaluated the sensory profile of PX and Muscat grapes, from three different harvests, which had been raisined either through ‘asoleo’ traditional overripening system or by means of a climatic chamber under temperature and moisture control. The results were very enlightening with regard to the considerable possibilities exhibited by the alternative raisining method. Particularly, the grapes from one of the harvests, which had been affected by rain falls during the days before their cropping, produced musts marked by more intense fungal or humidity notes as well as weaker fruity and aroma intensity when the grapes had undergone the ‘asoleo’ traditional overripening system than when the must was produced by means of a climatic chamber. The analysis of ochratoxin A (OTA) in the musts confirmed a 4-fold fungal contamination in the raisins obtained by ‘asoleo’ traditional overripening system (up to 28.8 g/kg) [63].

As already discussed, the sweet wines from the Jerez-Xérès-Sherry PDO require some degree of fermentation. Fermentation brings complexity and acidity, while balancing the intense sweet notes (fruit, raisin) that are predominant in wines that are simply the result of adding wine alcohol to the raisined grape must [58]. This was confirmed by Ruiz et al. [47], who carried out a study on the aromatic characterization of wines obtained from raisined PX grape musts as a result of the different degrees of fermentation. In another paper, Ruiz-Bejarano et al. [55] studied the volatile composition of sweet wines obtained from raisined Muscat musts under different vinification conditions, including as experimental variables the type of yeast (*S. cerevisiae* vs. *S. bayanus*), the fermentation temperature (room vs. chilled), the addition of ammonium phosphate nutrient, and the prefermentative pellicular maceration with pectolytic enzymes. According to their results, the concentrations of esters are favored by the addition of nutrients, by the practice of pellicular maceration with enzymes, and especially by the combination of these practices with the use of *S. bayanus* yeast. On the other hand, the concentration of acetates was encouraged by fermentation with *S. cerevisiae* at room temperature. Moreover, certain alcohols and aldehydes (1-hexanol, hexanal, benzaldehyde, 2-phenylethanol) increased their presence in those assays that included skin maceration with enzymes. From a sensory point of view [62,63], the sweet Muscat wines fermented at low temperature (< 10 °C) with *S. bayanus* yeast without nutrients and pectolytic enzymes, were characterized by intense citrus and floral notes and were the best rated, while the ones obtained using nutrients were granted the lowest scores. In a follow-up study [64], the same authors observed that the use of *S. bayanus* significantly decreased ethyl carbamate content in the wines—a compound declared to be carcinogenic—while the use of nutrients and pectolytic enzymes increased its content levels. PX wines, with their characteristic amino acid profile, presented lower concentrations of this compound than Muscat wines.

The high sugar concentration in raisin musts, as much as 400 g/L, causes some difficulties to the production of sweet wine. Espejo et al. [65] tested the use of pectolytic enzymes combined with prefermentative maceration to facilitate the pressing and improve must extraction yields. They succeeded to obtain wines with improved aromatic and taste characteristics. The use of osmo-resistant yeasts has been the subject of study of several researchers [49,53,66,67]. As an example of this, a study with *Torulaspora delbrueckii* [67], a yeast of low volatile acidity production capacity with concentrated musts, high aroma revealing capacity, but low alcohol resistance, produced wines with higher citrus notes, lower raisin notes, and better overall ratings than those fermented using *S. cerevisiae*. The concentrations of isoamyl alcohol, 2-phenylethanol, isobutyl alcohol, benzaldehyde, 2,2-diethoxyethyl benzene, and 2-phenylethyl isobutyrate increased, while those of ethyl butyrate, some acetates, and certain fatty acids decreased.

No work has been found in the literature on the aromatic evolution of natural sweet wines from the “Jerez-Xérès-Sherry” PDO during their aging by means of the Criaderas y Solera method. Only a limited number of related works have been found [57,68,69], but the production of the wine was carried out in a different way from those established for the “Jerez-Xérès-Sherry” PDO.

On the other hand, Ruiz-Bejarano et al. [54], analyzed the evolution of 51 volatile substances during the static aging of sweet wines made from PX and Moscatel grape musts from two different vintages in 30 L American oak barrels. With respect to aging time, several ethyl esters (ethyl 3-methylbutanoate, diethyl pentanedioate, and diethyl succinate) increased significantly, while ethyl decanoate and ethyl dodecanoate decreased, which is explained by hydrolysis and esterification phenomena. The acetates, n-butyl acetate, isoamyl acetate and phenylethyl acetate; the terpenes, nerol oxide, linalool, thymol, carvacrol and β-myrcene; the alcohols, 3-methyl-2-butanol and 1-hexanol; aldehydes such as benzaldehyde, nonanal, octanal, hexanal and 2-hexenal, 2-furaldehyde (originating from raisining) and 1,1,6-trimethyl-1,2-dihydronaphthale, increased significantly with aging time, probably as a result of their concentration. Some of the compounds detected and that mainly derive from contact with oak were eugenol, 4-ethylphenol, and 5-methylfuraldehyde. In a previous study [55], the same authors had investigated the effect of the type and time aging length on sweet Moscatel wines, by comparing aging in medium-toasted 30 L American oak barrels with the aging carried out through contact with chips of the same oak variety at doses of 4 g/L, as well as in the absence of wood. The levels of most compounds were affected by the presence or absence of wood and, to a large extent, also by the type of contact, i.e., barrel or chips. The sensory analyses [63], according to expectations, detected greater oak notes as aging time grew longer, although their intensity levels were higher in the cask-aged wines. It also established a clear preference for cask-aged wines over those aged in contact with chips, where an aromatic defect could be perceived. Cask-aging was confirmed as an improving agent and one that was particularly effective with grapes coming from less optimal harvests from a sensory point of view [62].

Ruiz et al. [47] studied the accelerated aging of sweet wines from raisined PX grapes in contact with American oak chips at doses of 1 and 2 g/L at 20 °C, together with other alternatives to the traditional method. They confirmed significant increases in 2,3-butanedione, isoamyl acetate, eugenol, vanillin, furfural, and 5-methylfurfural, and volatile phenols such as guaiacol, 4-ethylguaiacol, 4-ethylphenol, syringol and isoeugenol, as well as (E) and (Z) isomers of β-methyl-γ-octalactone.

Herrera et al. [45] monitored the static aging of a natural sweet PX wine in 16 L casks made of American, French and Spanish oak, as well as of chestnut wood. Some wood-derived compounds—such as eugenol, *trans*-whiskeylactone, benzaldehyde, or 5-methyl-2-furaldehyde among others—increased their concentrations with time, regardless of the botanical origin of the wood. The same happened with certain other compounds such as isobutyl acetate and isobutanol, which, as expected, also increased their concentration as a result of the evaporation of water through the wood pores.

## 4. Study of the Aroma of Sherry Vinegar

Sherry vinegar is a product resulting from the acetic fermentation of the wines produced in the Sherry region. It is produced and aged using traditional practices and must display certain organoleptic and analytical characteristics. Depending on the aging times to which the vinegars are subjected, the following are distinguished: Sherry Vinegar (six months minimum aging time), Reserva Sherry Vinegar (two years minimum aging time), and Gran Reserva Sherry Vinegar (10 years minimum aging time). In addition, there are also semi-sweet or sweet Sherry vinegars (depending on the amount of sugar), namely Pedro Ximénez Sherry Vinegar and Moscatel Sherry Vinegar, which have one of these types of sweet wines added during the aging process [8].

Vinegar aroma has been a subject of study for several decades, and Table 3 shows the different volatile compounds studied in Sherry vinegar, their sensory descriptors and the concentration ranges found in the bibliographic references.

In the 1990s, Blanch et al. [83] found no major differences between the volatile composition of the Sherry vinegars studied and other non-aged wine vinegars that were also considered in the study. However, it was observed in this work that the Sherry vinegars generally exhibited higher concentrations of most compounds and particularly of acetaldehyde, a compound that had already been found in previous studies also in aged vinegars [95]. Guerrero et al. [96] reached similar conclusions after analyzing Sherry vinegars and other unaged vinegars, which in this latter case had been produced by means of submerged culture acetification methods (quick acetification). This study was conducted according to the standardized analysis methods of the time. Morales et al. [84] showed that the use of NaOH or MgO to neutralize the high acetic acid content of vinegars prior to their analysis by gas chromatography significantly reduced the content of many of the volatile compounds that were originally present. Natera et al. [81] analyzed Sherry vinegars by means of solid phase microextraction (SPME) and the volatile compounds found in higher proportions were 2-methyl-1-propanol, 2- and 3-methyl-1-butanol, 3-hydroxy-2-butanone, 2-phenylethanol, isoamyl acetate, 2,3-butanediol, and isopentanoic acid.

**Figure 5:**
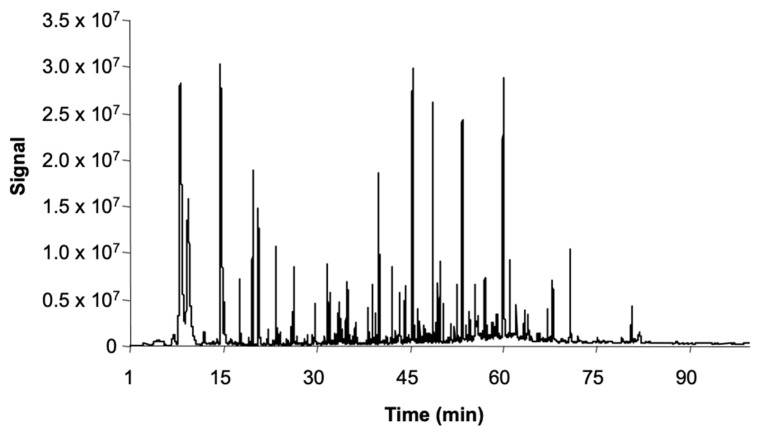
Example of chromatogram of a Sherry vinegar obtained by SBSE-GC-MS and retention times of some relevant compounds: ethyl isobutyrate (13.62); propyl acetate (13.99); isobutyl acetate (15.76); ethyl butyrate (16.84); ethyl isopentanoate (18.46); hexanal (18.70); isopentyl acetate (20.57); ethyl pentanoate (20.77); 1-butanol (21.84); 3-methyl-1-butanol (23.84); 2-methyl-1-butanol (24.12); ethyl hexanoate (24.65); hexyl acetate (25.80); 3-hydroxy-2-butanone (26.62); *cis* 3-hexenyl acetate (27.59); ethyl lactate (28.51); 1-hexanol (28.87); ethyl octanoate (31.87); 2-furaldehyde (32.87); benzaldehyde (35.15); isobutyric acid (36.84); 5-methyl-2-furaldehyde (36.95); butyric acid (38.89); isovaleric acid (40.28); diethyl succinate (40.58); α-terpineol (41.51); benzyl acetate (42.64); ethyl-2-phenyl acetate (44.59); phenylethyl acetate (45.95); hexanoic acid (46.57); benzyl alcohol (47.03); 2-phenylethanol (49.21), 2-ethyl hexanoic acid (50.17); octanoic acid (53.75); eugenol (57.21); decanoic acid (60.39); 5-hydroxymethyl-2-furaldehyde (68.90).

More recently, Guerrero et al. [97,98] were able to identify and successfully quantify 47 volatile compounds by means of stir bar sorptive extraction (SBSE). This extraction methodology prevented sample interferences and increased the analytical sensitivity (Figure 5). Callejón et al. [71] analyzed volatiles in Sherry and Rioja vinegars employing headspace sorptive extraction (HSSE) and observed that the latter allowed to determine up to 53 volatile compounds, with 5 of them detected for the first time in this matrix: ethyl 2-methylbutyrate, ethyl heptanoate, ethylfuroate, ethyl benzoate, and acetophenone. Even though the volatile profiles of both types of vinegars were qualitatively similar, the Sherry vinegars contained greater amounts of some of them, including ethyl butyrate, ethyl isovalerate, ethyl lactate, isovaleric acid, and 4-ethylphenol.

When comparing Sherry vinegars to vinegars from other Protected Designation of Origins (PDO), Ríos-Reina et al. [72] carried out a study for the discrimination of vinegars from the three vinegar Protected Designation of Origin (PDO)s in Spain (‘Sherry Vinegar’, ‘Vinegar of Condado de Huelva’, and ‘Vinegar of Montilla-Moriles’). Other authors evidenced that the volatile content in vinegar is influenced not only by the production process, which is similar for Sherry vinegars and vinegars from Huelva, but also by the raw material, in this case, the grape variety used, Palomino for Sherry PDO and Zalema for Huelva PDO, as well as by geographical factors associated to each PDO [73]. Other authors [90], compared Sherry vinegars to vinegars from Huelva PDO and from Montilla-Moriles PDO, and some of their volatile compounds, namely 1-heptanol, methyl nonanoate, 2-methylbutanoic acid, 2,2,6-trimethyl-cyclohexanone, trans-2-decenal, eucalyptol, and α-terpineol allowed the differentiation of Huelva PDO vinegars from those produced under the Sherry PDO and Montilla-Moriles PDO, while diacetyl, acetoin, ethyl 3-ethoxypropanoate, 2- and 3-heptanone, 2-methyl-1-hexadecanol, 1-octen-3-ol, p-cresol, and camphene allowed to differentiate the vinegars from the Montilla-Moriles PDO. Moreover, Sherry PDO vinegars could be differentiated by their β-damascenone, 5-hydroxymethylfurfural, 3-heptanol, trans-2-hexen-1-ol, and trans-2-hexen-1-yl acetate contents. All of this not only corroborates the conclusions reported by previous studies, but also demonstrates that PDO vinegars can be classified based on their volatile profiles.

These differences were also observed in the studies carried out by means of Fourier transform mid-infrared spectroscopy (FTIR) with attenuated total reflectance (ATR) on Sherry and Huelva vinegars, both PDOs from Andalusia. These vinegars are produced following similar oenological practices that include different periods of aging in oak wood using the well-known Criaderas y Solera system. The authors concluded that aging in oak wood by means of Criaderas y Solera presented a series of bands in the region of 1500–900 cm^-1^ of the spectrum that enabled their differentiation according to the aging time of the vinegars from both PDOs. Aging in wood led to significant changes in the ATR-FTIR spectra due to a greater presence in the vinegars of compounds such as acetic acids, alcohols, esters, and ethers [99]. This spectroscopic technique has also been successfully applied to the differentiation of vinegars derived from different raw materials and production processes, including Sherry vinegars [100]. The percentage of successful classification achieved was similar to that obtained based on their volatile content.

Casale et al. [91] who observed that the determination of the spectral fingerprint of 17 Sherry vinegars together with other vinegars of different nature or origin (white wine, red wine, balsamic vinegar, apple vinegar, etc.) by Heaspace mass spectrometry without a previous chromatographic separation, allowed to differentiate them from the rest of the vinegars. Other study allowed the differentiation of the Sherry vinegars studied from other white and red wine vinegars, as well as from apple and balsamic vinegars, based only on 14 compounds among which eugenol (2-methoxy-4-prop-2-enyl-phenol), furfural (2-furancarboxaldehyde), several organic acids (isobutyric acid, nonanoic acid, etc.), some aldehydes, and esters (benzyl acetate, ethyl benzeneacetate, and ethyl benzoate) were the most relevant [77].

Benito et al. [101] carried out the characterization and differentiation of 66 vinegars from wines from the PDO “Rioja” and 18 from the Sherry PDO on the basis of different analytical parameters including glycerol and acetoin content along with other parameters such as organic acids, pH, acidity, Cu, Fe, etc. For this purpose, they used both classical statistical techniques (cluster analysis, principal component analysis) and others of later development, such as neuronal networks. These authors observed that, although a significant variability was observed in both groups of vinegars in terms of the parameters considered, given the wide range of aging times applied to the vinegars, they could be clearly differentiated by means of either set of chemometric techniques.

However, not only the raw material used which could determine the volatile composition, but also aging process, environmental conditions, microbiological activity could also induce different volatile profiles. The differences found between the different types of vinegars, including Sherry vinegars, and according to the studies that have been considered, seem to be due to both the starting raw material and the special and specific circumstances under which the production processes are carried out. In order to differentiate between relevant and irrelevant factors in the production of Sherry vinegar, Morales et al. [88] carried out a study in which they addressed the acetification stage by means of a submerged culture, as a factor that could determine the composition of the vinegar obtained, as opposed to the raw material used. The results revealed very significant changes in the volatile profile of the product as a consequence of the acetification process, even though the polyphenolic compounds content was not altered by this process. Therefore, the raw material used was considered to be the predominant factor. Durán et al. [102] also studied the changes that take place in the volatile composition over the acetification process of Sherry vinegars and succeeded to correlate it with the FTIR signal obtained.

**Figure 6:**
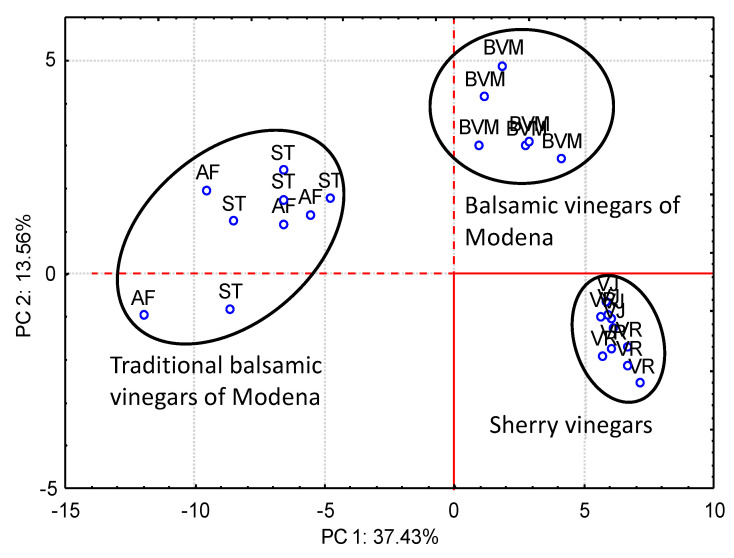
Differentiation of Sherry vinegars from Italian vinegars, based on their volatile content. According to Marrufo et al. [79] with modifications. AF: Affinato Traditional Balsamic Vinegar of Modena; ST: Stravecchio Traditional Balsamic Vinegar of Modena; BVM: Balsamic Vinegar of Modena; VJ: Sherry Vinegar; VR: Reserva Sherry Vinegar.

Chinnici et al. [70] studied the possibility of differentiating between Sherry and Modena vinegars from different categories (traditional Modena balsamic vinegar “extravecchio”, traditional Modena balsamic vinegar “affinato”, and Modena balsamic vinegar). In their study they reported 93 volatile compounds detected and identified by Solid Phase Extraction (SPE). The study revealed the differentiation between the different vinegars on the basis of several parameters such as the extent of Maillard reactions, alcoholic, or non-alcoholic fermentation, or the length of wood aging. In the same line of work, Marrufo et al. [79] using in this case SBSE-GC-MS, obtained a 100% separation between traditional Modena balsamic vinegars, Modena balsamic vinegars, and Sherry vinegars on the basis of furanes, terpenes, acetates, and esters (Figure 6). Durán et al. [94] observed a significant differentiation between Sherry vinegars and Modena balsamic vinegars according to their aldehydic compounds content.

For an in-depth characterization of the volatile profile of Sherry vinegars and its contribution to the perceived aroma, Aceña et al. [74] conducted a study by Gas Chromatography-Olfactometry (GC-O) on extracts from commercial Sherry vinegars obtained by HS-SPME. Among the 37 odorants found, some of them presented OAVs (odor activity values) greater than 1 (ethyl isovalerate, ethyl isobutyrate, isoamyl acetate, isovaleric acid, 2-phenylethanol, 4-ethylguaiacol, isobutyric acid, 2-phenylethyl acetate, and 4-ethylphenol), which suggests their significant contribution to the vinegar aroma.

Callejón et al. [75] were able to detect 108 aromatic notes by GC-O in Sherry vinegars, and identified 64 of them. In addition, they found that the mixture of compounds whose aroma most resembled the aroma of Sherry vinegar was a combination of diacetyl, ethyl acetate and sotolon. A more recent study has investigated the olfactometric profile of Sherry vinegars (dry and sweet Pedro Ximénez), together with vinegars from other Spanish denominations of origin (Montilla-Moriles and Condado de Huelva) and concluded that the most abundant aromas in the Sherry vinegars identified by GC-O belonged to the “grassy vegetal” family, while the “spicy” family of compounds was more characteristic of the sweet PX vinegars [80]. These authors were able to satisfactorily correlate the values obtained by GC-MS-O with those obtained by sensory analysis. Therefore, it seems clear that the volatile compound composition of vinegar is closely related to the aroma perceived by sensory analysis, which is why the latter discipline has become in recent years a clear complement to the analysis of the aromatic profile of oenological products in general, and of vinegars in particular.

The first methodological approach to sensory analysis applied to Sherry vinegars was carried out by González-Viñas et al. [103]. These authors conducted a study in which they determined the taste group thresholds (geometric mean of the individual best-estimate thresholds (BETs)) in organic acid solutions and in vinegars. This study demonstrated that the aromatic profile of the sample has an influence on the perception of the different descriptors, as was the case with the acid descriptors. On the other hand, Tesfaye et al. [104] developed a methodology for the sensory analysis of vinegars and applied it to the characterization of Sherry vinegar aroma after aging in wood. In that study, they observed that, a significant improvement in the quality of the vinegar aroma could be perceived after the first six months of aging. Later, these authors perfected the sensory analysis methodology applied to Sherry vinegars and succeeded in considerably reducing the deviations between judgments and the increment in the number of descriptors [105]. To date, the aroma of sherry vinegar has been characterized in detail from the sensory point of view [82] and the descriptors “glue”, “wood”, and “pungent” are typical of this type of vinegar, regardless of the aging method applied. On the other hand, the descriptors “raisin” and “alcohol/liquor” tend to be more characteristic of longer-aged vinegars (Gran Reserva), while the descriptor “wine character” at higher values is generally associated to younger Sherry vinegars [82].

As we have established, the aging process has a strong influence on the aromatic profile of oenological products. In the case of vinegar, it has been proven that there are numerous chemical and biochemical transformations that take place during the aging process, and that are similar to those that occur during the aging of Sherry wines either during their biological or oxidative aging. This fact has made of this stage a target for many studies on Sherry vinegar, as we have already seen. Thus, Palacios et al. [16] reported significant increases in acetic acid and other compounds such as acetoin, due to water loss by evaporation. However, other compounds, such as higher alcohols, decreased as a consequence of the synthesis of acetates. Similarly, the high concentration level of the residual alcohol that can be found in Sherry vinegars, together with their high acidity, favors higher concentrations of ethyl acetate to be developed during the aging process in comparison with other types of vinegars. This fact has been corroborated by other authors [86], who described significant rises in ethyl acetate concentrations during the aging of Sherry vinegars with a residual alcohol content of around 2%. These authors also described increments in other compounds—such as methyl acetate, methanol, diacetyl or γ-butyrolactone—that took place during the aging of Sherry vinegars. However, it cannot be ignored that, as already mentioned, other factors—such as the acetification system used—may modify, even more than the actual aging process, the volatile content of Sherry vinegars [85].

The type of wood used for aging also seems to have an impact on the volatile composition of Sherry vinegars. American oak (*Quercus alba*) is the most commonly wood used, but other types of wood such as French oak (*Quercus petraea*), Spanish oak (*Quercus pyrenaica*), or chestnut (*Castanea sativa*) have also been employed [92]. It has been demonstrated that chestnut wood provides a significantly different volatile profile with respect to that obtained from oak woods, and that Spanish oak and French oak woods provide a similar content of volatile compounds in aged vinegars. Moreover, from the sensory point of view, it has been observed that French oak wood provides highly favorable organoleptic characteristics to aged vinegars, while Spanish oak wood generates vinegars that are quite similar to those traditionally aged in American oak casks [92].

On the other hand, the aging of Sherry vinegar in wood containers is a lengthy and costly process that is susceptible of shortening. However, in order to preserve the typicity of this product, it is essential to verify that the volatile profile of the product obtained by accelerating methods does not differ from that obtained by traditional aging procedures. Hence, some studies have dealt with the sensory profile of vinegars aged in an accelerated manner using American and French oak chips [106]. The authors concluded that the differences between the samples were mostly due to the pungency of the samples rather than to the character provided by oak wood. Generally speaking, Sherry vinegars elaborated in a traditional way showed higher scores for the attributes studied: aromatic intensity, richness in aroma, ethyl acetate, woody odor, wine character, pungent sensation, coconut, vanillin, clove odor, and general impression. In addition, woody odor was very similar for both samples, traditionally aged and infused with oak chips. On the other hand, Durán Guerrero et al. [107] presented a method to accelerate the aging of Sherry vinegars by the joint application of micro-oxygenation and wood shavings while trying to resemble the natural aging process that takes place in wooden casks. Using an oxygen dose of 70 mL/L/month and 5 g/L of American oak chips they were able to obtain, in just 14 days, vinegars with a volatile profile similar to those aged by traditional methods in 105 days (86% time reduction). More recently, Jiménez-Sánchez et al. [78] used a combination of micro-oxygenation, wood shavings and ultrasound energy to further accelerate the aging process of Sherry vinegars. In this case, different types of wood were used (American, French, and Spanish oak), and it was observed that Spanish oak provided a greater amount of volatile compounds. In addition, with the combined use of ultrasound, wood shavings and micro-oxygenation, the vinegars obtained in just 4 days, had similar volatile profiles to those of vinegars aged by the traditional method for 6 months.

**Figure 7:**
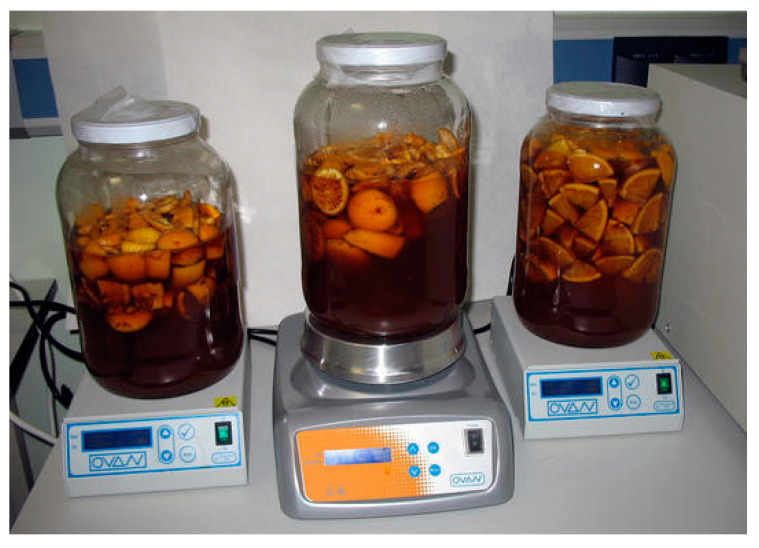
New products derived from Sherry vinegar: maceration with citrus fruits.

Finally, it is worth mentioning that, although Sherry vinegar is a product of ancient tradition, it is also open to innovation and has recently been used in the development of new products. Aroma is a key factor in the elaboration of such new products derived from Sherry vinegar, and has therefore been studied in different occasions. For example, the effect that the maceration with peels from different fruits (orange, lemon, lime, grapefruit, strawberry) exerts on the aroma of Sherry vinegar has been studied (Figure 7) and a product with a marked fruity character has been obtained by using peel concentrations at 200 g/L and 3-day maceration time [89]. From a sensory point of view, descriptors ‘fruity’, ‘sweet’, and ‘aroma intensity’ were directly correlated with olfactory impression, which means that the preference of the vinegars was mainly based on these three descriptors. Moreover, the descriptors that allowed the best discrimination among vinegars macerated with different fruits were fruity, citric, and sweet.

In a subsequent study, the maceration time was reduced to a few minutes by applying accelerating energies, such as microwaves or ultrasound [93]. The aroma of this type of product obtained by maceration was studied by GC-MS-O, and it was observed that there was a significant increase mainly in compounds with ‘floral’ aromas. Vinegars macerated presented high content in alcohols, aldehydes, and terpenoids, and from a sensory point of view, the lowest values of floral, greasy and citric categories were obtained for vinegars without maceration [87]. Another example of innovation concerning Sherry vinegar is that proposed by Marrufo-Curtido et al. [76] where dietary fiber from citrus fruits was added to the vinegars with an increase in the sensory descriptor ‘citrus’ observed in the final product. In addition, these fiber-enriched vinegars were very highly valued from a sensory point of view. Finally, Sherry vinegars have also been used in the development of other novel products by adding small quantities to fruit juices in order to produce soft drinks [108]. The character provided by the addition of vinegar improved the sensory properties of the fruit juices, which were favorably rated in a subsequent consumers’ survey. Based on the olfactory and gustatory impression, and purchase intent, the acetic beverages made from peach and pineapple juices were the most appreciated, followed by apple juice, while those obtained from orange juice were the least preferred by consumers.

## 5. Study of the Aroma of Sherry Brandy

Sherry brandy displays certain characteristics that differentiate it from other aged spirits. Such characteristics derive from their aging according to the dynamic system known as Criaderas y Solera, and from the requirement to age in preconditioned 500–600 L capacity oak casks, mostly American oak [109]. According to their minimum average aging time, Sherry brandies are classified into three categories: Solera Brandy (6 month minimum aging time), Solera Reserva Brandy (1 year minimum aging time), and Solera Gran Reserva Brandy (3 year minimum aging time).

The composition of a Sherry brandy is determined by:

(1) The grape variety from which the initial wine distillate is obtained (mainly Airén, Palomino, and Pedro Ximénez grapes) [110,111];
(2) The fermentation and production conditions of the base wine [112];
(3) The processing and nature of the initial distillate, a mixture containing varying quantities of *holanda* (low-grade spirit), medium-grade spirits and distillates (high-grade spirit), with at least 50% of the total ethanol content coming from medium and low grade spirits [19,113,114];
(4) The origin and conditioning of the wood cask, i.e., the type of oak and its toasting intensity [115,116];
(5) The preconditioning of the cask with wine, i.e., the type of wine that it has previously contained and for how long [13];
(6) The previous length or frequency of use of the barrel, i.e., whether it is used to produce brandy for the first time after its preconditioning with wine or it has been used several times to hold and produce brandy [117].

All of these factors have an impact on the physicochemical and organoleptic characteristics of Sherry brandies and provide them with a rich and varied aroma. However, with regard to their aromatic profile scarce bibliography is available. Table 4 presents the volatile compounds determined in Sherry brandy, their sensory descriptors and the concentration ranges found in the bibliographic references.

Durán et al. [118,119], after the analysis of 48 Sherry brandies, emphasized the quantitative importance of isoamyl alcohol, 2-methyl-1-butanol, benzaldehyde, diethyl succinate, 2-phenylethanol, octanoic acid, decanoic acid, lauric acid, ethyl decanoate, and ethyl octanoate, with concentration levels above mg/L. Several of the compounds identified seemed to increase with aging time, although only ethyl esters, 2-phenylethyl acetate, linalool and eugenol did so significantly. A number of the compounds identified, such as ethyl laureate, ethyl myristate, ethyl palmitate, and lauric acid, were derived from the initial distillate, and their starting acids (lauric acid, myristic acid, caprylic acid, …) may also be present, since they are the precursors of the esterification reactions with ethanol that give place to the appearance of the above mentioned esters. Some of the compounds may also have their origin in the wood itself (caprylic acid, myristic acid, or palmitic acid, among others) or in the wine preconditioning process [13] such as ethyl lactate or ethyl succinate. The furfuryl compounds may also come from two sources, since they are generated in the thermal processes during the distillation, but also during the toasting of the cask wood and then transferred to the spirits [10,120]. Other compounds such as vanillin and certain coumarins have also been identified [121].

Multivariate statistical techniques have been used to determine the discrimination accuracy between the three types of Sherry brandies based on their aromas. The results pointed towards a clear differentiation of Solera from both Solera Reserva and Solera Gran Reserva [119], where the last ones showed a widely dispersed pattern. These results are in agreement with those from other works on the polyphenolic composition of brandies in which the discrimination of the intermediate Solera Reserva only reached 57% [10]. The reason for this characteristic pattern could be the lack of a minimum aging time. Nevertheless, when an electronic nose that allows the analysis of global aromatic profiles was used, higher discrimination percentages were achieved for the different categories of brandies [122].

Although the concentration of some of these polyphenolic compounds over time tends to increase mainly due to either wood extraction or water losses during the aging process, it has been demonstrated that the brandies that are aged in old casks—i.e., casks that were not used for the first time for this purpose—continue to evolve and gradually improve the complexity of their aroma [117]. This takes place at an evidently slower rate mostly due to the Criaderas y Solera system which involves a periodic supply of air that favors oxidative phenomena. We should point out the long aging times for Solera Gran Reserva Sherry brandies which is generally in the order of 20 years or more in currently commercialized brandies [123].

**Figure 8:**
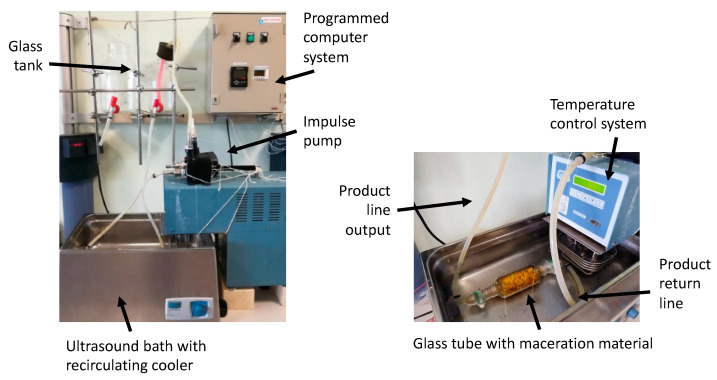
Ultrasound system for the accelerated aging of oenological products [124].

As previously mentioned, the aging of Sherry brandy is considered its most characteristic production stage, i.e., the one that provides it with its distinctive character, and since the associated costs are rather high, as it was seen for other Sherry products above, considerable interest has been shown to investigate alternative methods to accelerate the process while preserving the product’s chemical and sensory profile. Among such methods, those that use wood chips and ultrasound as the accelerating energy, with or without the addition of air, are the ones that have gained most of the attention [124,125,126], since they can shorten aging times by 6 to 18 times (Figure 8).

These are the tools that have been used at laboratory or pilot plant scale to evaluate the suitability of different varietal spirits to be aged as Sherry brandy [110]. It has been concluded that the effect of aging is different depending on the grape variety, thus the aroma profile of the worst rated young brandies improved (as occurred with Ugni Blanc and Corredera), while the aged Muscat of Alexandria and Garrido brandies were awarded lower sensory ratings compared to their unaged samples. In the same study, the spirits that had been made from Jaén Blanco and Zalema grapes were the most appreciated, both young and aged, which were equally characterized by clear fruity notes and high aromatic intensities. This accelerated aging system has also been used to determine the potential of woods from different botanical origin (American, French, and Spanish oak, chestnut and cherry) for the aging of brandies [127], and to evaluate the use of Colombard, Moscatel, Palomino fino, Pedro Ximénez, and Zalema varietal holandas distilled by means of a rotary evaporator [114] to produce brandy. Some of the products obtained were rated high by a tasting panel.

## 6. Final Remarks

As can be seen from all this research, the uniqueness of Sherry oenological products, in terms of their aromatic composition, is determined both by the raw materials used and by each and every one of the significantly conditioning factors in their production processes. This also includes a number of environmental factors and, in particular, the aging stage. All of these factors contribute to the highly distinctive aroma displayed by Sherry wines, vinegars, and brandies and make of them the superior oenological products that are internationally acclaimed. Moreover, although aroma of Sherry products has been widely studied to date, due to the complexity of these special products, further innovation in analytical methodologies and advanced instrumentation is still needed. The reliable analysis of volatile compounds may contribute to a better knowledge and quality control of Sherry products, and therefore to meet the high levels of consumer demand, in an increasingly competitive sector.

## Figures and Tables

**Table 1:** Volatile compounds identified in dry Sherry wines, sensory descriptors, concentration ranges, and bibliographic references

Volatile Compounds	Sensory Descriptors	Concentration (mg/L)	References Fino	References Amontillado	References Oloroso
*Carbonyls*					
Acetaldehyde	Overripe apple	85–545	[31,32,33,34,35,36]	[32,37]	[32,38]
Acetoin	Butter	0.011–74	[31,32,33,34,35,36]	[32,37]	[32]
Benzaldehyde	Bitter almond/cherry	0.013–0.076	[33,36]		[39]
2,3-Butanedione	Butter-cookie	0.170–2.1	[33,34,36]	[37]	[38]
Furfural	Sweet/woody/almond/baked/bread	0.179–7.14	[32]	[32]	[32]
β-Ionone	Balsamic/rose/violet/berry/phenolic	0.062	[32,35]	[32]	
Neral	Sweet/citrus/lemon peel		[33]		
Octanal	Herbaceous	0.090–0.390	[32,33,34,35,36]	[37]	
*Acids*					
Butanoic acid	Cheese/butter	0.607–14.6	[31,32,33,34,35,36]	[32,37]	[32,38]
Decanoic acid	Rancid	0.004–0.370	[31,33,36]		[39]
Dodecanoic acid	Mild fatty/coconut/bay oil		[33,36]		
Hexanoic acid	Fatty/sweat/cheese	0.635–2.39	[31,32,33,34,35,36]	[32]	[32]
Isobutanoic acid	Acidic/cheese/dairy/buttery/rancid	2.2–22.1	[31,33,36]		
Isobutyric acid	Acidic/cheese/dairy/buttery/rancid	0.002–4.58		[32]	[32,39]
3-Methylbutanoic acid	Cheese	1.5–679	[31,32,33,35,36]	[32,37]	[38]
Nonanoic acid	Waxy/cheesy/dairy	0.003–0.011			[39]
Octanoic acid	Fatty/waxy/rancid/oily/cheesy	0.001–1.6	[31,33,34,36]		[39]
*Alcohols*					
Benzyl alcohol	Floral/rose/phenolic/balsamic	0.045–3.3	[31,32,33,36]	[32]	[32]
1-Butanol	Fusel oil/sweet/balsam/whiskey	0.001–19.9	[31,32,33,34,35,36]	[32]	[32,39]
2-Butanol	Sweet/apricot	1.1–4.4	[31,32,33,35,36]	[32]	[32]
2,3-Butanediol	Fruity/creamy/buttery		[33,36]		
1-Decanol	Fatty/waxy/floral	0.124–1.26	[32,33,35,36]	[32]	[32]
3-Ethoxy-1-propanol		0.250–0.490	[31,33,35]		
1-Heptanol	Musty/pungent/leafy green/apple/banana	0.300–0.870	[33]	[32]	[32]
Hexanol	Fusel oil/fruity/alcoholic/sweet/green	0.001–2.5	[31,32,33,35,36]	[32]	[32,39]
E-3-Hexenol	Green/cortex/floral/oily/earthy	0.055–0.085	[31,32,35]		
Z-3-Hexenol	Green/grassy/melon rind	0.055–0.085	[31,32,33,35]		
Isoamyl alcohols	Vinous/solvent	0.020–444	[31,32,33,34,35,36]	[32,37]	[32,38]
Isobutanol	Vinous/solvent	25.7–102	[31,32,33,34,35,36]	[32,37]	[32]
Isopropyl alcohol	Alcohol/musty/woody	1.4–2.7	[31]		
Methanol	Slight alcoholic		[33,36]		
2-Methyl-1-butanol	Roasted/fruity/fusel oil/alcoholic/wine/whiskey				[39]
2-Methyl-1-pentanol		0.020–0.090	[32]	[32]	[32]
3-Methyl-1-pentanol	Pungent/fusel oil/brandy/wine/cocoa	0.110–18	[31,32,33,35,36]	[32]	[32]
4-Methyl-1-pentanol	Nutty	0.029–0.135	[31,32,33,36]		[32]
1-Octanol	Waxy/green/citrus/aldehydic/floral/coconut		[33,36]		
1-Pentanol	Pungent/fermented/bready/yeasty/fusel oil/winey/solvent	0.060–102	[32,33]	[32]	[32]
Phenethyl alcohol	Rose	0.003–99	[31,32,33,34,35,36]	[32,37]	[32,38,39]
Propanol	Alcoholic/fermented/musty/yeasty/apple/pear	12.3–16.3	[31,33,35,36]		
*Volatile phenols*					
4-Ethylguaiacol	Toasted/clove	0.002–0.740	[32,33,34,35,36]	[32,37]	[32,39]
4-Ethylphenol	Smoke/phenolic/creosote	0.004–0.094	[33,36]		[32,39]
Eugenol	Cinnamon/clove	0.002–0.477	[31,32,33,34,35,36]	[32,37]	[32,39]
Guaiacol	Phenolic/smoke/spice/vanilla/woody	0.280–0.434			[39]
Methyleugenol	Spicy/cinnamon/clove/musty/waxy/phenolic	0.157			[32]
*Esters*					
Butyl acetate	Fruity/solvent/banana	0.091–0.161	[33]		[32,39]
Diethyl malate	Brown sugar/sweet/wine/fruity/herbal	0.800–23.6	[31,32,33,35,36]	[32]	[32]
Diethyl succinate	Mild fruity/cooked apple/ylang	0.001–55.4	[31,32,33,35,36]	[32]	[32,39]
Ethyl acetate	Pineapple/varnish	13.9–260	[31,32,33,34,35,36]	[32,37]	[32]
Ethyl benzoate	Fruity/dry/musty/sweet/wintergreen	0.180–0.215	[32]		[32]
Ethyl butanoate	Banana/apple	0.172–3.5	[31,32,33,34,35,36]	[37]	[32,38,39]
Ethyl decanoate	Sweet/waxy/fruity/apple/grape/oily/brandy	0.22			[32]
Ethyl furoate	Balsamic		[33,36]		
Ethyl heptanoate	Fruity/pineapple/brandy/rum/wine	0.021–0.109	[32,35]	[32]	[32]
Ethyl hexanoate	Almond/apple	0.078–0.280	[31,33,34,35,36]	[37]	[38,39]
Ethyl 3-hydroxybutanoate	Fruity/green grape/tropical	0.030–0.747	[31,33,35,36]		
Ethyl 3-hydroxyhexanoate	Rubber		[33]	[37]	
Ethyl isobutanoate	Apple/pineapple	0.028–1.660	[31,32,33,34,35,36]	[32,37]	[32,38]
Ethyl isovalerate	Fruity/sweet/apple/pineapple/tutti frutti	0.001–0.009			[39]
Ethyl lactate	Raspberry/milky	12–854	[31,32,33,34,35,36]	[32,37]	[32,38]
Ethyl laurate	Sweet/waxy/floral/soapy/clean	0.024–0.140	[32,35]	[32]	[32]
Ethyl myristate	Mild waxy/soapy	0.099–0.119	[32,33,35,36]	[32]	
Ethyl octanoate	Pear	0.008–1.3	[31,32,33,34,35,36]	[32,37]	[39]
Ethyl propanoate	Sweet/fruity/rum/juicy fruit/grape/pineapple	0.109–1.92	[31,32,33,35,36]	[32]	[32]
Ethyl palmitate	Mild waxy	0.042–0.070	[32,35]	[32]	
Ethyl pyruvate	Fruity/sweet/rum	0.081–0.201	[31,32,33,35,36]	[32]	[32]
Ethyl valerate	Sweet/fruity/apple/pineapple/green/tropical	0.001–0.010			[39]
Hexyl acetate	Green/fruity/sweet/fatty/fresh/apple/pear	0.001–0.008			[39]
Hexyl hexanoate	Green/sweet/waxy/fruity/berry	0.247			[32]
Hexyl lactate	Sweet/floral/green/fruity	1.1			[32]
Isoamyl acetate	Banana	0.050–0.855	[31,33,34,36]	[37]	[38,39]
Isoamyl laurate	Winey/alcoholic/fatty/creamy/yeasty/fusel oil	0.357		[32]	
Isobutyl acetate	Sweet/fruity/banana	0.025–0.137	[31,33]		[32,39]
Isobutyl isobutanoate	Fruity tropical/fruit pineapple/grape skin/banana	0.066		[32]	
Isobutyl lactate	Faint buttery/fruity/caramel	0.034–0.242	[32,33,35,36]		[32]
Methyl acetate	Solvent/fruity/winey/brandy/rum	6.6			[32]
Methyl butanoate	Strawberry/butter	0.486–4.86	[33,34,36]	[37]	[32,38]
Monoethyl succinate	Odorless		[33,36]		
Phenethyl acetate	Flowers	0.100–1.1	[31,33,36]	[37]	[38]
Phenethyl octanoate	Sweet/waxy/slightly cocoa/caramel/winey/brandy	0.190–0.275	[32,33,36]	[32]	
Propyl acetate	Solvent/fusel oil/sweet/fruity	0.042–0.162	[31,32,33,36]	[32]	[32]
Propyl butanoate	Pungent/rancid	0.112–0.150	[32,35]	[32]	
*Terpenes*					
β-Citronellol	Rose	0.280–1.33	[31,32,33,35,36]	[32]	
Farnesol	Sweet/floral	0.282–5.79	[32,35]	[32]	[32]
Linalool	Citrus/orange/floral/terpy/waxy/rose	0.009–0.032	[31]		[38]
Nerol	Floral/green	0.151–0.176	[32,35]	[32]	
E-Nerolidol	Floral/green/citrus/woody/waxy	0.076–0.213	[32,35]	[32]	[32]
Z-Nerolidol	Waxy/floral	0.696	[32,33,36]		
4-Terpineol	Pine	0.777	[32]		
α-Terpineol	Pine/woody/resinous/lemon/citrus	0.006–0.015	[32,33,35]		[39]
*Lactones*					
γ-Butyrolactone	Creamy/oily	0.004–40.8	[31,32,33,34,35,36]	[32]	[32,39]
γ-Decalactone	Peach	0.043	[32,33,34,35,36]		
Pantolactone		0.470–5.22	[31,32,33,35,36]	[32]	[32]
Sotolon	Walnut/cotton candy/curry	0.100–0.670	[33,34,36]	[37]	[38]
cis-Whiskeylactone	Burnt/wood/vanilla/coconut	0.009–0.410	[31,34,36]	[37]	[38,39]
trans-Whiskeylactone	Sweet/spicy/coconut/vanilla		[33]		[39]
*Miscellaneous*					
Methionol	Cooked potato/cut hay	0.063–3.4	[31,32,33,34,35,36]	[32,37]	[32,38]
p-Cymene	Citrus/terpene/woody/spice		[33,36]		
1,1-Diethoxyethane	Green fruit/liquorice	8.4–58.8	[31,32,33,34,35,36]	[32,37]	[32,38]

**Table 2:** Volatile compounds identified in natural sweet wines, sensory descriptors, and concentration ranges reported in the bibliographic references

Volatile Compounds	Sensory Descriptors	Concentration (mg/L)	References
*Alcohols*			
(E)-2-Hexenol	Herbaceous/green/green tomato	0.001–0.36	[46,47,48,49,50,51]
2,3-Butanediol (levo/meso)	Ripe fruit/butter	0.001–4015.0	[47,49,52,53]
2-Butanol	Vinous/medicinal	0.003–0.12	[46,47,48,49,50,51]
2-Methylbutanol	Roasted/fruity/ alcoholic/fusel oil/ wine/whiskey	1.40–1.66	[45]
2-Phenylethanol	Rose/talc/honey	0.12–78.88	[45,47,48,49,51,52,53,54,55]
2-phenylethyl alcohol	Rose/honey	0.002–25.91	[46,50,56,57]
3-Ethoxypropanol	Overripe pear	0.30–17.37	[47,49]
3-Hexenol (E/Z)	Herbaceous/green/grass	0.001–0.079	[46,47,48,49,52,56]
3-Methyl-2-butanol			[54]
3-Methylpentanol	Pungent/fusel oil/brandy/wine/cocoa	0.022–0.030	[47]
Benzyl alcohol	Roasted/toasted/disifectant/fruity/walnut/floral/rose/phenolic/balsamic	0.001–0.772	[45,46,47,48,49,50,51,52]
Butanol	Vinous/medicinal	0.001–1.76	[45,46,47,48,49,50,51]
Heptanol	Oily	0.006–0.037	[46,57]
Hexanol	Cut grass/resinous/herbaceous/wood	0.001–1.02	[45,46,47,48,49,50,51,54,55,57]
Isoamyl alcohols	Solvent/cake/fusel alcohols/nail polish/ripe fruit	0.003–146.72	[46,47,48,49,50,51,52,53,55,56,57]
Isobutanol	Alcohol/solvent/vinous/nail polish	0.003–40.90	[45,46,47,48,49,50,51,53,56,57]
Methanol	Solvent/pungent fruity	57.5–163.0	[49,53,57]
Pentanol	Bitter almond/synthetic	0.001–0.014	[49,51]
Propanol	Fusel alcohol/ripe fruit	8.4–88.0	[49,53,57]
*Aldehydes*			
(E)-2-Hexenal	Herbaceous	0.012–0.308	[48,51,55]
2-Hexanal			[54]
3-Methylbutanal	Ethereal/aldehydic/chocolate/peach/fatty	0.094	[56]
Acetaldehyde	Stewed apple/pungent	13.29–347.0	[49,53,56,57]
Benzaldehyde	Roasted/bitter almond/nutty/smoky	0.003–0.151	[45,46,47,48,49,50,54,55]
Decanal	Soapy/green lemon		[57]
Hexanal	Fatty/herbaceous/green apple	0.004–0.444	[46,47,48,49,50,52,54,55]
Nonanal	Waxy/aldehydic/rose/orange peel fatty		[54,55]
Octanal	Herbaceous	0.046–0.127	[47,54,55]
Phenylacetaldehyde		0.068	[56]
*Ketones*			
2,3-Butanedione	Buttery/ripe fruit/yogurt/cake	0.004–5.07	[46,47,48,49,50,51,56,57]
2,3-Pentanedione	Buttery/cream/cake	0.004–0.435	[46,47,48,50,57]
2-Octanone	Floral/over ripe fruit	0.002–0.022	[51]
6-Methyl-5-hepten-2-one			[54,55]
Acetoin	Buttery/cream/sour yogurt/sour milk	0.070–1228.52	[46,47,48,49,50,51,53,57]
*Furans*			
2-Furaldehyde	Fusel alcohol/cake/burnt/almond/ripe fruit/toasted bread/incense/floral	0.001–5.002	[45,46,47,48,49,50,51,52,54,55,56,57]
5-Hydroxymethyl-2-furaldehyde	Rancid/toasted	0.003–102.40	[45,51]
5-Methyl-2-furaldehyde	Toasted/bitter almond/cake/burnt/caramel	2.4	[45,46,47,48,49,50,51,52,54,55,57]
Ethyl 2-furoate	Balsamic		[57]
Furfuryl alcohol	Varnish	0.005–0.023	[47,49,57]
*Acids*			
2-Ethyl-hexanoic acid			[54,55]
2-Methylbutanoic acid	Rancid	0.003–0.009	[48]
3-Methylbutanoic acid	Lactic/rancid/cheese	0.001–2.495	[45,46,47,48,50,56,57]
Acetic acid	Fatty	3.31–4.08	[52]
Butanoic acid	Aged cheese/rancid	0.003–0.627	[46,47,48,49,50,56]
Decanoic acid	Rancid/cheese/wax/plasticine	0.005–0.185	[45,47,49,51,52,54,55,57]
Dodecanoic acid	Fatty/coconut/bay		[54,55]
Hexadecanoic acid	Waxy/fatty		[54,55]
Hexanoic acid	Cheese/rancid	0.030–0.069	[49]
Isobutanoic acid	Cheese/rancid/fat	0.003–5.623	[45,49]
Nonanoic acid	Waxy/dirty/cheese/dairy	0.011–0.033	[45,54,55]
Octanoic acid	Rancid/cheese/fatty	0.002–0.506	[45,47,48,49,51,52,54,55,57]
Propanoic acid	Fat	0.080–1.371	[49]
Tetradecanoic acid	Waxy		[54,55]
*Esters*			
2-Phenylethyl acetate	Fruity/honeyed/floral/rose	0.001–0.094	[46,47,48,49,50,52,54,55,56,57,58]
2-Phenylethyl hexanoate		0.007–0.015	[47]
2-Phenylethyl octanoate	Cocoa/caramel/winey/brandy		[57]
3-Methylpropyl acetate		0.037	[56]
4-Methyl-2-pentyl acetate		0.181	[52,55]
Benzyl acetate	Floral/fruity/jasmine/fresh	0.0416	[45]
Butyl acetate	Solvent/fruity/banana	0.016–0.154	[45,47,54,55]
Butyl lactate			[57]
cis-3-Hexenyl acetate		0.001–0.002	[45]
Diethyl malate	Green	0.003–0.531	[47,49,51]
Diethyl pentanedioate			[55]
Diethyl succinate	Overripe fruit/lavender	0.101–1.76	[45,47,49,52,54,55,57,58]
Ethyl 2-methylbutanoate		0.0041	[56]
Ethyl 2-methylpentanoate			[56]
Ethyl 2-methylpropanoate		0.054	[56]
Ethyl 3-hydroxybutanoate	Grape/green apple/marshmallows	0.005–0.062	[47,49,57]
Ethyl 3-methylbutanoate		0.0075	[45,54,55,56]
Ethyl 3-methylpentanoate		0.001	[56]
Ethyl 4-methylpentanoate		0.001	[56]
Ethyl acetate	Pineapple/varnish/balsamic/fruity/solvent/pungent/glue	0.031–113.33	[46,47,48,49,50,52,53,57]
Ethyl benzoate	Fruity/medicinal/wintergreen/anise	0.002–0.005	[49,57]
Ethyl butanoate	Banana/pineapple/strawberry	0.012–0.386	[45,52,54,55,56,57,58]
Ethyl cyclohexanoate			[56]
Ethyl decanoate	Synthetic/rancid	0.015–0.162	[52,54,55,57,58]
Ethyl dihydrocinnamate		0.001	[56]
Ethyl dodecanoate	Waxy/floral/soapy/clean	0.077–0.106	[54,55,58]
Ethyl furoate	Plum/floral	0.0001	[49]
Ethyl heptanoate	Strawberry/banana	0.005–0.046	[46,47,58]
Ethyl hexadecanoate		0.008	[54,55,58]
Ethyl hexanoate	Banana/green apple	0.005–0.147	[45,47,49,54,55,56,57,58]
Ethyl isobutanoate	Apple/pineapple	0.002–3.869	[45,58]
Ethyl lactate	Lactic/yogurt/strawberry/raspberry/buttery	0.001–93.8	[46,47,48,50,51,52,57]
Ethyl octadecanoate			[54,55]
Ethyl octanoate	Pineapple/pear/soapy/banana	0.002–0.174	[45,49,52,54,55,56,57,58]
Ethyl pentanoate	Fruity/apple/pineapple/green/tropical	0.005–0.071	[45,52]
Ethyl propanoate	Banana/apple	0.005–0.152	[46,47,58]
Ethyl succinate	Toffee/coffee	0.029–70.0	[47,49]
Ethyl tetradecanoate	Mild waxy/soapy	0.002	[54,55,58]
Hexyl acetate	Apple/pear/banana/floral	0.001–2.14	[45,46,47,48,50,52,57]
Isoamyl acetate	Banana	0.008–0.019	[49,54,55]
Isoamyl butanoate	Banana/fruity	0.012–0.089	[47,48]
Isobutyl lactate	Faint buttery/fruity/caramel		[57]
Methyl acetate	Solvent/fruity/winey/brandy/rum	0.064–0.085	[58]
Methyl butanoate	Strawberry/butter		[57]
Methyl octanoate		0.001	[52]
*Terpenes*			
4-Terpineol	Moldy	0.002	[48,54,55]
Carvacrol	Thyme		[54,55]
Farnesol	Floral/fruity/balsamic/clove	0.002–0.080	[46,47,48,50,54,57]
Geranial	Citrus	0.002–0.078	[46,47,50]
Geraniol	Floral/fruity/rose/waxy/citrus		[54]
γ-Terpineol		0.034–2.99	[52]
Linalool	Muscat/rose/lavender	0.006–1.62	[52,54,55,56,57]
Linalool oxide			[54,55]
Nerol	Citrus/magnolia	0.013	[47,54,55]
Nerol oxide			[54,55]
Nerolidol	Floral/green/citrus/woody/waxy		[54,55]
p-Cymene	Fresh/citrus/lemon/woody/spicy	0.23–0.58	[52]
Thymol	Herbal/thyme/phenolic/medicinal/camphor		[54,55]
α-Terpineol	Lily/cake	0.004–0.016	[45,47,48,54,55]
β-Citronellol	Rose		[54,55]
β-Myrcene			[54,55]
*Lactones*			
4-Caprolactone	Herbaceous/coconut	0.001–0.005	[49]
γ-Butyrolactone	Cake/caramel/fruity/empyreumatic/coconut/toasted	0.003–37.90	[45,46,47,48,49,50,51,57]
γ-Decalactone	Peach/coconut	0.001–0.129	[46,47,48,49,50,58]
γ-Heptalactone	Fruity/coconut/herbaceous/caramel	0.001–0.120	[46,47,48,50]
γ-Hexalactone	Cake/fruity/peach	0.003–0.023	[47,48]
γ-Nonalactone	Over-ripe fruit	0.015–0.372	[51,58]
γ-Pentalactone	Cut hay	0.002–0.006	[49]
Pantolactone	Toasted bread/smoked	0.065–0.190	[47,49,57]
Sotolon	walnut/cotton candy/curry	0.176	[56]
cis-Whiskeylactone	Burnt/wood/vanilla/coconut	0.011–0.028	[47,56,57]
trans-Whiskeylactone	Spicy/coconut/vanilla	0.004–0.049	[45,47,57]
*Mercaptans*			
2-Methyl-3-furanthiol	Fried	0.035	[56]
3-Mercaptohexanol	Green/lemon		[56]
4-Mercapto-4-methyl-2-pentanone	Broom/cat urine/black currant sprout		[56]
Dimethyl disulphide (DMDS)		0.0098	[56]
Methional	Boiled vegetables/oxidized	0.02	[56]
Methionol	Cooked potato/cut hay	0.001–0.070	[46,47]
*Methoxypyrazines*			
3-Isobutyl-2-methoxypyrazine	Green pepper/asparagus/potato		[56]
3-Isopropyl-2-methoxypyrazine			[56]
3-sec-Butyl-2-methoxypyrazine			[56]
*Miscellaneous*			
1,1,6-Trimethyl-1,2-dihydronaphthalene (TDN)	Gasoline		[54,55]
1,1-Diethoxyethane	Green fruit/licorice/cake/fruity/over-ripe fruit	0.023–4.795	[46,47,48,50,51,57]
β-Damascenone	Fruity/rose/plum/grape/raspberry	0.01	[56]

**Table 3:** Volatile compounds identified in Sherry vinegars, sensory descriptors, and concentration ranges reported in the bibliographic references

Volatile Compounds	Sensory Descriptors	Concentration (mg/L)	References
*Acetates*			
Benzyl acetate	Sweet/floral/fruity/jasmine/fresh	0.013–0.224	[70,71,72,73,74,75,76,77,78]
Bornyl acetate	Woody/pine/herbal cedar/spice		[79]
2,3-Butanediol diacetate			[79,80]
n-Butyl acetate	Solvent/fruity/banana	0.1–2.8	[71,73,75,77,80,81,82]
Ethyl acetate	Fruity/sweet/weedy/green	0.1–3.9	[16,72,73,75,79,80,82,83,84,85,86,87,88]
Ethyl 2-phenyl acetate	Sweet/floral/honey/rose/balsamic/cocoa	25–132	[70,71,73,74,79,80,81,82,87,89]
Geranyl acetate	Floral/rose/lavender/ green/waxy		[79,89]
(E)-2-Hexen-1-ol acetate	Green/fruity		[79,90]
(Z)-3-Hexen-1-ol acetate	Green/fruity/banana/apple/grassy	0.01–0.03	[73,78,79,80,91]
Hexyl acetate	Fruity/green apple/banana/sweet	0.007–0.09	[71,72,73,75,78,79,83,87,92,93]
Isoamyl acetate	Sweet/fruity/banana	2.7–16.3	[71,72,73,74,75,78,79,80,82,83,86,93]
Isobutyl acetate	Sweet/fruity/banana	1.0–4.3	[71,72,73,75,78,79,80,82,83,87]
Methyl acetate	Sweet/fruity	0.011–0.05	[71,72,75,82,84,85,86,88,90]
4-Methyl-2-pentyl acetate	Sweet/fruity/banana		[79,87,89,93]
2-Methyl-1-propyl acetate	Sweet/fruity/apple banana	9.97	[84,89]
Neryl acetate	Floral/rose/citrus/pear		[79]
3-Oxobutan-2-yl acetate	Pungent/sweet/creamy/buttery		[90]
Phenylethyl acetate	Floral/rose/sweet/honey/fruity/tropical	0.5–4.8	[70,71,72,74,75,79,80,81,82,83,87,93]
Phenyl methyl acetate	Sweet/floral/honey/ spicy/waxy/almond		[79,94]
1,2-Propanediol diacetate	Fruity/acetic		[79]
Propyl acetate	Solvent/fruity/fusel/raspberry/pear	0.06–0.2	[71,72,73,75,78,82,85]
*Esters*			
Diethyl succinate	Mild fruity/cooked apple	0.007–2.44	[70,71,72,73,75,78,79,80,81,82,83,84,86,87,88,89,92,93]
Dihydroxymethyl jasmonate			[79]
Ethyl benzoate	Fruity/dry musty/sweet	0.006–0.013	[71,72,75,77,80]
Ethyl butanoate	Sweet/fruity/tutti frutti	0.05–0.3	[71,72,73,74,75,78,79,80,83,84,87,93]
Ethyl ciclohexanoate			[80]
Ethyl decanoate	Sweet/waxy/fruity/apple/grape/oily	0.008–0.054	[72,73,79,81,83,89,93]
Ethyl dodecanoate	Sweet/waxy/floral/soapy/clean		[72]
Ethyl-3-ethoxypropanoate			[72,90]
Ethyl formate	Green/alcohol/rose/cognac	24.3–194	[85,86,88]
Ethyl heptanoate	Fruity/pineapple/cognac/rum/wine		[80]
Ethyl hexanoate	Sweet/fruity/pineapple/waxy/green banana	0.05–75	[70,71,72,73,75,78,79,81,83,87,92,93]
Ethyl hydrogensuccinate			[70,72]
Ethyl isobutyrate	Sweet/ethereal/fruity/alcoholic/fusel	0.006–1	[71,72,73,74,75,77,78,82,92]
Ethyl isovalerate	Fruity/sweet/apple/pineapple/tutti frutti	0.03–1.1	[71,72,73,74,75,78,79,80,82,87,89,92]
Ethyl lactate	Sharp/tart/fruity/buttery/butterscotch	0.007–63	[70,71,73,82,85,86,88]
Ethyl levulinate	Sweet/fruity/floral/ berry/green pineapple/rhubarb		[79]
Ethyl 2-methyl butanoate	Sharp/sweet/green/apple/fruity	0.07–0.15	[71,72,73,74,75,79,80,82,83,87,89,93]
Ethyl 3-methylpentanoate	Pineapple/fruity/tropical		[80]
Ethyl nonanoate	Fruity/rose/waxy/rum/wine/tropical		[80]
Ethyl octanoate	Fruity/wine/waxy/sweet/apricot/banana/brandy/pear	0.02–0.05	[71,72,73,75,79,80,83,89,92]
Ethyl propanoate	Fruity/banana/pineapple	0.6–1.5	[71,72,75,79,80,82,83]
Ethyl vanillate	Phenolic/burnt/smoky/powdery/metallic		[70,79]
Ethyl valerate	Sweet/fruity/apple/pineapple/green	0.002–0.67	[71,72,73,75,78,79,81,82,92,93]
Isobutyl isothiocyanate	Green		[79]
Methyl butyrate	Fruity/apple/sweet/ banana/pineapple		[80]
Methyl hexadecanoate	Oil/waxy/fatty		[89,90]
Methyl hexanoate	Fruity/pineapple		[79]
Methyl nonanoate	Sweet/fruity/pear/waxy/tropical/wine		[90]
Methyl 9-octadecanoate			[90]
Methyl salicylate	Mint		[70,72,77,79,87]
*Acids*			
Acetic acid	Sharp/pungent/sour/vinegar		[16,72,79,80,83,87,89,93]
Benzoic acid	faint balsam/urine		[70,79,80,89]
Butanoic acid	Sharp/cheesy/rancid/butter		[70,72,77,79,80,83]
Decanoic acid	Unpleasant/ rancid/sour/fatty	0.03–0.5	[70,71,72,73,74,75,78,79,81,82,83,87,89,92,93]
Dodecanoic acid	Fatty/coconut/bay		[90,93]
2-Ethylhexanoic acid			[72]
Formic acid	Pungent/vinegar		[79]
Heptanoic acid	Rancid/sour/cheesy/sweat	0.10–0.15	[71,77]
Hexadecanoic acid	Waxy/fatty		[70,72,79,89]
9-Hexadecenoic acid			[79,89]
Hexanoic acid	Sour/fatty/sweat/cheese	1.3–2.2	[70,71,72,73,75,77,79,80,81,82,83]
(4-Hexyl-2,5-dioxo-2,5- dihydro-3-furanyl) acetic acid			[90]
Isobutyric acid	Acidic/sour/cheese/dairy/buttery/rancid	0.06–0.15	[72,73,74,78,80,90,92]
Isopentanoic acid	Stinky feet/sweaty/cheese	49–60	[70,71,72,73,74,75,78,79,80,81,82,83,87,89,90,92]
Nonanoic acid	Waxy/dirty/cheese/dairy	0.01–0.04	[71,72,77,78,79,87,92,93]
Octadecanoic acid	Fatty/waxy		[79]
9-Octadecenoic acid			[79]
Octanoic acid	Fatty/waxy/rancid/oily/vegetable/cheesy	0.7–2.6	[70,71,72,73,75,78,79,80,81,82,83,87,89,92]
Oleic acid	Faint fatty/waxy		[79]
Pentadecanoic acid	Waxy		[72,79,89]
Pentanoic acid	Acidic/sweaty/rancid		[70,72]
Phenylacetic acid	Sweet/honey/floral/honeysuckle/sour/waxy/		[70]
Propanoic acid	Pungent/acidic/cheesy/vinegar		[70,72,80]
Sorbic acid			[72]
Tetradecanoic acid	Waxy		[70,72,79,89]
*Alcohols*			
2-Acetoxy-1-propanol			[70]
Benzyl alcohol	Floral/rose/phenolic/balsamic	81–1980	[70,71,75,78,79,80,81,82,83,92,93]
Borneol	Balsamic/camphoreous/herbal/woody		[79]
2,3-Butanediol	Fruity/creamy/buttery	353–95	[80,81,85,88,91]
1-Butanol	Fusel/oily/sweet/ balsamic/whiskey		[70,93]
Butoxyethoxyethanol			[70]
Ethanol	Alcoholic/medical/strong	1.03–9000	[72,75,80,82,85,86,88]
3-Ethoxy-1-propanol	Fruit		[70,79]
4-Ethyl resorcinol			[76]
γ-Eudesmol (2-naphthalene methanol)	Waxy/sweet		[77,93]
Eugenol	Sweet/spicy/clove/woody	0.01–0.1	[70,71,73,75,77,78,92]
Fenchyl alcohol	Camphoreous/pine/woody/dry/rooty/sweet/lemon		[79,89]
1-Heptanol	Musty/leafy/violet/herbal/green/sweet/woody/peony		[90]
2-Heptanol	Fresh/lemon/grass/herbal/sweet/floral/fruity/green		[90]
3-Heptanol	Herbal		[90]
1-Hexanol	Ethereal/fusel/oily/fruity/alcoholic/sweet/green	0.002–0.4	[71,72,73,78,87,92]
2-Hexanol	Chemical/winey/fruity/fatty/cauliflower		[89,93]
trans 2-Hexen-1-ol	Fresh/green/leafy/fruity/unripe banana		[73,90]
cis 3-Hexen-1-ol	Fresh/green/grassy/foliage/vegetable/ herbal/oily	0.04–0.05	[71,75,80,82,87]
Methanol	Alcoholic	11–67	[16,75,82,85,86,88]
2-Methyl-1-butanol	Roasted winey onion/fruity/fusel/alcoholic/whiskey	560–13,000	[71,72,73,74,75,78,79,81,82,83,84,85,86,88,89,92,93]
3-Methyl-1-butanol	Fusel/alcoholic/pungent/cognac/fruity/banana	5000–60,000	[16,70,71,72,73,74,75,79,80,81,82,83,84,85,86,88,89,90,93]
2-Methyl-1-hexadecanol			[90]
2-Methyl-1-propanol	Winey/whiskey	3.5–14.3	[71,73,74,75,80,81,82,85,86,88,93]
1-Nonanol	Fresh/clean/fatty/floral/rose/orange/dusty/wet/oily		[89,90]
Phenylethyl alcohol	Sweet/floral/fresh/rose	0.013–27.1	[70,71,72,74,75,78,79,80,81,82,83,84,85,86,87,88,89,90,92,93]
1-Propanol	Alcoholic/fermented/fusel/tequila/musty/ sweet/fruity/apple/pear	0.66–13.1	[75,80,82,84,86,88]
Propano-1,2,3-triol		3200–21,600	[16]
Vanillyl alcohol	Sweet/creamy/vanilla/caramellic/cracker/milky/		[80]
*Phenols*			
4-Acetyl-2-methylphenol			[79]
2/4-Ditertbutyl phenol			[79]
4-Ethylguaiacol	Spicy/smoky/bacon/phenolic/clove	0.6–2.9	[70,71,72,73,74,78,79,80,81,89,92]
4-Ethylphenol	Phenolic/smoky	0.02–1.6	[70,71,73,74,75,78,79,80,81,82,87,89,90,92,93]
Guaiacol	Phenolic/smoky/spicy/vanilla/woody	0.009–0.016	[75,78,80,82,90]
4-Methylguaiacol	Spicy/clove/vanilla/phenolic/medicinal/leathery		[80]
Phenol	Phenolic/plastic/rubber		[79,83]
*Terpenes*			
Camphene	Woody/herbal/fir needle/camphor		[89,90]
Citronellene	Floral/rose/herbal/citrus/		[79]
β-Citronellol	Floral/leathery/waxy/ rose/citrus		[87,89]
Cymene	Fresh/citrus/lemon/woody/spicy		[79]
Eucalyptol	Eucalyptus/herbal/camphoreous/medicinal		[79,90]
Geraniol	Sweet/floral/fruity/rose/waxy/citrus		[79,89]
Limonene	Pine/herbal/peppery		[79,87,89]
Linalool	Citrus/floral/sweet/bois de rose/woody/green/blueberry		[79,87,89]
trans p-Mentha-2,8-dienol			[90]
Nerol	Sweet/citrus/magnolia		[79,89,93]
Perillaldehyde	Fresh/green/oily/grassy/fatty/minty/cherry		[79]
Safranal	Fresh/herbal/phenolic/metallic/rosemary/tobacco/spicy		[90]
δ-Selinene			[79]
γ-Terpinene	Oily/woody/lemon/ lime/tropical/herbal		[79]
4-Terpineol	Peppery/woody/earthy/musty/sweet		[79,87,89]
α-Terpineol	Pine/lilac/citrus/woody/floral	0.007–0.1	[71,73,77,78,79,87,89,90,92,93]
β-Terpineol	Pungent/earthy/woody		[89]
Thymol	Herbal/thyme/phenolic/medicinal/camphor		[89]
*Aldehydes*			
Acetaldehyde	Pungent/ethereal/aldehydic/fruity	5–61	[16,75,84,85,88]
Benzaldehyde	Strong/sharp/sweet/bitter/almond/cherry	0.05–1070	[70,71,72,73,75,77,78,80,81,83,84,87,89,90,91,92,93,94]
Butanal	Pungent/cocoa/musty/green/malty/bready		[94]
2-Butenal	Floral		[94]
Cuminaldehyde (4-(1-methylethyl)-benzaldehyde)	Spicy/cumin/green/herbal		[77]
Decanal	Sweet/aldehydic/waxy/orange peel/citrus/floral		[94]
trans 2-Decenal	Fatty/orange/rose/aldehydic/floral/green		[90]
Dodecanal	Soapy/waxy/aldehydic/citrus/green/floral		[94]
Heptanal	Fresh/aldehydic/fatty/green/herbal/cognac/		[94]
Hexanal	Fresh/green/fatty/aldehydic/grassy	0.009–0.05	[71,73,94]
Isobutyraldehyde	Fresh/aldehydic/floral/green		[94]
Isovaleraldehyde	Aldehydic/chocolate/ peach/fatty		[79]
2-Methylbutanal	Musty/cocoa/coffee/nutty		[74,94]
3-Methylbutanal	Ethereal/aldehydic/chocolate/peach/fatty		[74,94]
3-Methylpropanal			[94]
Nonanal	Waxy/aldehydic/rose/orange peel/fatty		[74,87,89,93,94]
(E)-2-Nonenal	Fatty/green/cucumber/aldehydic/citrus		[74,80,94]
Octanal	Aldehydic/waxy/citrus/orange peel/green/fatty	0.011–0.014	[74,79,87,90,93,94]
3-Octanal			[89]
Pentanal	Fermented bready/fruity/nutty/berry		[94]
Propanal	Earthy/alcoholic/winey/whiskey/cocoa/nutty		[94]
Undecanal	Waxy/soapy/floral/aldehydic/citrus/green/fatty/fresh laundry		[94]
Vanillin	Sweet/vanilla/cream/chocolate	2.5–4.4	[70,71,75,80]
*Furanic componds*			
5-Acetoxymethyl-2-furaldehyde	Baked bread		[72,73,79]
2-Acetyl-2,5-dimethylfuran			[79]
2-Acetylfuran	Sweet/balsamic/almond/cocoa/caramellic/coffee	0.6 × 10^−5^–1.7 × 10^−5^	[70,79,82,90]
2-Acetyl-5-methylfuran	Musty/nutty/hay/coconut/milky		[70,73,79]
5-Ethoxymethylfurfural			[79]
Ethyl furoate		0.03–0.2	[71,72]
Furfural	Sweet/woody/almond/fragrant/baked bread	0.1–2.2	[70,71,72,73,75,77,78,79,81,83,87,91,92,94]
Furfuryl alcohol	Alcoholic/chemical/musty/sweet/caramel/bread/coffee	0.3–1.04	[70,71,75,80,82]
5-Hydroxymethylfurfural	Fatty/buttery/musty/waxy/caramellic		[70,72,79]
5-Methylfurfural	Sweet/caramellic/bready/coffee	0.005–0.02	[70,71,72,75,78,79,80,92]
1-(5-Methyl-2-furyl)-1-propanone			[79]
*Ketones*			
Acetoin	Sweet/buttery/creamy/dairy/milky/fatty	0.28–708	[16,70,71,72,73,75,77,79,80,81,83,84,85,86,88,90,93]
Benzophenone	Balsamic/rose/metallic/geranium		[79,90]
2,3-Butanodione	Buttery/sweet/creamy/pungent/caramellic	17–42	[71,72,74,75,80,86,90,91]
β-Damascenone	Sweet/fruity/rose/plum/grape/raspberry/sugar		[80,90]
3-Heptanone	Green/fatty/fruity		[90]
2-Heptanone	Fruity/spicy/sweet/herbal/coconut/woody		[90]
Hydroxyacetone	Pungent/sweet/caramellic/ethereal	5.34–70	[86]
3-Hydroxy-3-methyl-2-butanone			[70]
α-Ionone	Sweet/woody/floral/violet/tropical/fruity	0.018–0.038	[81]
β-Ionone	Floral/woody/sweet/ fruity/berry/tropical		[80]
Isovalerone	Green/fruity/metallic/pineapple/banana		[79]
5-Methyl-3-hexanone			[79]
3-Nonanone	Fresh/sweet/jasmin/spicy/herbal/fruity		[72]
1-Octen-3-one	Herbal/mushroom/earthy/musty/dirty		[74,80]
1-(2,3,6-Trimethylphenyl)-3-buten-2-one			[79]
*Lactones*			
γ-Butyrolactone	Creamy/oily/fatty/caramellic	0.005–0.38	[70,71,75,78,82,84,85,86,88,92]
δ-Decalactone	Fresh/oily/waxy/peach/coconut/buttery/sweet		[79]
δ-2-Decenolactone			[79]
γ-Dodecalactone	Fatty/peach/sweet/metallic/fruity		[80]
γ-Heptalactone	Sweet/coconut/nutty/caramellic/		[79]
δ-Laurolactone			[79]
α-Methyl-γ-crotonolactone			[79]
Pantolactone	Cotton/candy		[80]
Solerone			[70]
Sotolone	Sweet/caramellic/maple/sugar burnt/sugar/coffee	0.748	[75,80]
cis-Whiskeylactone	Coconut/toasted/nutty/burnt	0.1–1.5	[70,71,75,79,80,82]
trans-Whiskeylactone	Coconut/toasted/nutty/celery/burnt	0..07–0.3	[70,71,75,78,79,82,92]
*Enolic derivatives*			
Cyclotene	Sweet/caramel/maple/sugar/coffee/woody		[70,79,90]
2,3-Dihydro-3,5-dihydroxy-6-methyl-4H-pyran-4-one			[79]
3-Ethyl-2-hydroxy-2-cyclopenten-1-one	Sweet/caramellic/maple		[70,79]
Cyclotene	Sweet/caramel/maple/sugar/coffee/woody		[70,79,90]
*Miscellaneous*			
2-Butyl-4-methyl-1,3-dioxolane	Nutty/fatty		[90]
Cadalene (1,6-dimethyl-4-(1-methylethyl)-naphthalene)			[77]
Cyclotetradecane			[79]
Dibutyl formamide			[79]
N,N-Dimethylformamide	Slight amine		[79]
Methyl styrene			[79]
N-(3-Methylbutyl) acetamide			[79]
Pentadecane	Waxy		[79]
Styrene	Sweet/balsamic/ floral/plastic		[79]
Tetradecane	Mild waxy		[79]
Tridecane			[79]
1,1,6-Trimethyl-1,2- dihydronaphthalene (TDN)	Gasoline	4.4 × 10^−5^–10.5 × 10^−5^	[72,82,90]
Vitispirane	Floral/fruity/earthy/woody		[89]

**Table 4:** Volatile compounds identified in Sherry brandy, sensory descriptors and concentration ranges reported in the bibliographic references.

Volatile Compounds	Sensory Descriptors	Concentration (mg/L)	References
*Alcohols*			
2-Butanol	Vinous/medicinal	1.8	[117]
2-Methylbutanol	Roasted/fruity/fusel oil/alcoholic/wine/whiskey	80.9–181.8	[117,118,119]
2-Phenylethanol	Rose/talc/honey	4.99–22.4	[118,119]
2-Phenylethyl alcohol	Rose/honey	2.16–2.52	[117]
3-Hexenol (E/Z)	Herbaceous/green/grass	0.238–2.245	[118,119]
Butanol	Vinous/medicinal	7.92–9.36	[117]
Hexanol	Cut grass/resinous/herbaceous/wood	3.99–10.44	[117,118,119]
Isoamyl alcohols	Solvent/cake/fusel alcohols/nail polish/ripe fruit	193–678	[117,118,119]
Isobutanol	Alcohol/solvent/vinous/nail polish	119.88–133.92	[117]
Methanol	Solvent/pungent fruity	238.32–245.16	[117]
*Aldehydes*			
Acetaldehyde	Stewed apple/pungent/	78.84–86.76	[117]
Benzaldehyde	Roasted/bitter almond/nutty/smoky	2.91–35.3	[118,119]
*Furans*			
2-Furaldehyde	Fusel alcohol/cake/almond/toasted bread/incense/floral	0.19–14.54	[10,116,117,120]
5-Hydroxymethyl-2-furaldehyde	Rancid/toasted	0.072–87.09	[10,116,117,120]
5-Methyl-2-furaldehyde	Toasted/bitter almond/cake/burnt/caramel	0.062–1.94	[10,116,117,120]
*Acids*			
Acetic acid	Fatty	210.1–307.6	[116]
Decanoic acid	Rancid/cheese/wax/plasticine	5.12–15.1	[118,119]
Dodecanoic acid	Fatty/coconut/bay	1.51–7.18	[118,119]
Octanoic acid	Rancid/cheese/fatty	0.007–13.4	[118,119]
*Esters*			
2-Phenylethyl acetate	Fruity/honeyed/floral/rose	0.013–0.119	[118,119]
Diethyl succinate	Overripe fruit/lavender	0.071–5.40	[118,119]
Ethyl 2-methylbutanoate		0.103–0.241	[119]
Ethyl 2-methylpropanoate		0.064–0.454	[118]
Ethyl acetate	Pineapple/varnish/balsamic/fruity/solvent/pungent/glue	134.28–236.52	[117]
Ethyl butanoate	Banana/pineapple/strawberry	0.327–14.9	[118,119]
Ethyl decanoate	Synthetic/rancid	0.64–4.93	[117,118,119]
Ethyl dodecanoate	Sweet/waxy/floral/soapy/clean	0.160–1.08	[117,118,119]
Ethyl heptanoate	Strawberry/banana	0.057–0.104	[118,119]
Ethyl hexadecanoate	Mild waxy	1.44	[117]
Ethyl hexanoate	Banana/green apple	0.46–1.79	[117,118,119]
Ethyl isopentanoate	Fruity/sweet/apple/pineapple/tutti frutti	0.090–0.443	[118,119]
Ethyl lactate	Lactic/yogurt/strawberry/raspberry/buttery	48.24–50.76	[117]
Ethyl nonanoate	Fruity/rose/waxy /rum/wine/tropical		[118,119]
Ethyl octanoate	Pineapple/pear/soapy/banana	0.63–5.4	[117,118,119]
Ethyl pentanoate	Sweet/fruity/apple/pineapple/green	0.041–0.398	[118,119]
Ethyl succinate	Toffee/coffee	3.96–7.2	[117]
Ethyl tetradecanoate	Mild waxy/soapy	0.36	[117]
Hexyl acetate	Apple/pear/banana/floral	0.0004–0.003	[118,119]
Isoamyl octanoate		0.002–0.018	[118,119]
Isoamyl acetate	Sweet/fruity/banana	0.101–1.098	[118,119]
(E)-Methyl-2-octenoate		0.0007–0.0027	[118,119]
Methyl decanoate		0.001–0.007	[118,119]
*Terpenes*			
Linalool	Muscat/rose/lavender	0.053–0.590	[118,119]
Nerolidol	Floral/green/citrus/woody/waxy	0.002–0.004	[118,119]
α-Terpinene		0.0017	[118,119]
α-Terpineol	Lily/cake	0.007–0.097	[118,119]
*Volatile phenols*			
4-Ethylguaiacol	Spicy/smoky/bacon/phenolic/clove	0.046–0.210	[118,119]
Eugenol	Cinnamon/clove	0.007–0.071	[118,119]
Vanillin	Vanilla	0.13–5.94	[10,116,117,120]
*Miscellaneous*			
1,1-Diethoxyethane	Green fruit/licorice/cake/fruity/overripe fruit	105.84–115.56	[117]
β-Damascenone	Fruity/rose/plum/ grape/ raspberry	0.001–0.084	[118,119]
